# Improved DNA Extraction and Illumina Sequencing of DNA Recovered from Aged Rootless Hair Shafts Found in Relics Associated with the Romanov Family

**DOI:** 10.3390/genes13020202

**Published:** 2022-01-23

**Authors:** Odile Loreille, Andreas Tillmar, Michael D. Brandhagen, Linda Otterstatter, Jodi A. Irwin

**Affiliations:** 1 Federal Bureau of Investigation Laboratory, DNA Support Unit, Quantico, VA 22135, USA; mdbrandhagen@fbi.gov (M.D.B.); jairwin@fbi.gov (J.A.I.); 2 Department of Forensic Genetics and Forensic Toxicology, National Board of Forensic Medicine, SE-587 58 Linkoping, Sweden; andreas.tillmar@rmv.se; 3 Department of Biomedical and Clinical Sciences, Faculty of Medicine and Health Sciences, Linköping University, SE-582 25 Linkoping, Sweden; 4 Federal Bureau of Investigation Laboratory, Trace Evidence Unit, Quantico, VA 22135, USA; lmotterstatter@fbi.gov

**Keywords:** Romanov family, next generation sequencing (NGS), hybridization capture, mitochondrial DNA, heteroplasmy, single nucleotide polymorphism (SNP), kinship analyses, biological sexing, ancient DNA

## Abstract

This study describes an optimized DNA extraction protocol targeting ultrashort DNA molecules from single rootless hairs. It was applied to the oldest samples available to us: locks of hairs that were found in relics associated with the Romanov family. Published mitochondrial DNA genome sequences of Tsar Nicholas II and his wife, Tsarina Alexandra, made these samples ideal to assess this DNA extraction protocol and evaluate the types of genetic information that can be recovered by sequencing ultrashort fragments. Using this method, the mtGenome of the Tsarina’s lineage was identified in hairs that were concealed in a pendant made by Karl Fabergé for Alexandra Feodorovna Romanov. In addition, to determine if the lock originated from more than one individual, two hairs from the locket were extracted independently and converted into Illumina libraries for shotgun sequencing on a NextSeq 500 platform. From these data, autosomal SNPs were analyzed to assess relatedness. The results indicated that the two hairs came from a single individual. Genetic testing of hairs that were found in the second artifact, a framed photograph of Louise of Hesse-Kassel, Queen of Denmark and maternal grandmother of Tsar Nicholas II, revealed that the hair belonged to a woman who shared Tsar Nicholas’ maternal lineage, including the well-known point heteroplasmy at position 16169.

## 1. Introduction

The fate of the Romanov family was considered one of the greatest mysteries of the 20th century and generates interest and speculation to this day. While official written records are scarce, reports indicate that Tsar Nicholas and his family were held prisoner and shot in July 1918 by a Bolshevik firing squad in the Ipatiev House (Ekaterinburg, Russia). According to accounts, the bodies were then buried in a superficial, hastily dug pit [1,2].

In 1991, nine skeletons were recovered from a shallow grave in Ekaterinburg and tentatively identified by Russian authorities as the Tsar, the Tsarina, three of their five children, and four family retainers. Subsequent DNA testing confirmed the identification of the five Romanovs [3]. The testing included mitochondrial DNA (mtDNA) analysis of the purported Tsarina and three of her daughters. Their mtDNA profile was compared to the profile of one of the Tsarina’s living grandnephews, Prince Philip, Duke of Edinburgh, who, belonged to the same maternal lineage. Testing also included biological sex determination and autosomal short tandem repeats (STR) analyses and all the DNA results established the presence of a family group consistent with the Romanovs. Additionally, mtDNA testing of the putative remains of the Tsar yielded a sequence that was consistent with the mtDNA profiles of two living maternal relatives of the Tsar as well as the mtDNA profile of the exhumed remains of the Tsar’s brother, Grand Duke George Romanov [3,4]. After 16 years following the discovery of the first grave, the missing son and daughter were accounted for when a few small bone fragments were discovered close to the site of the previous recoveries. Anthropological as well as mitochondrial and nuclear DNA analyses of these bone fragments confirmed the presence of the two Romanov children that were missing from the first grave [5,6].

**Figure 1:**
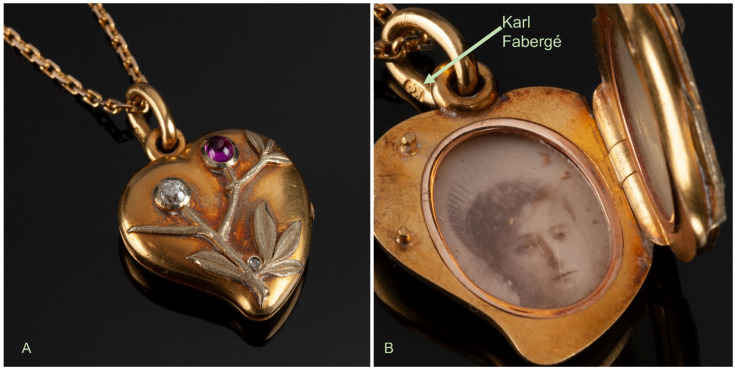
(**A**) Closed Fabergé locket showing precious stones. (**B**) The open locket showing a picture of Tsarina Alexandra Feodorovna Romanov. The stamped initials from Karl Fabergé can be seen on the jump ring. Photographed by Richard Walker. Courtesy of the Russian History Museum.

Recently, the DNA Support Unit (DSU) of the FBI laboratory was made aware of two artifacts containing biological material believed to be associated with the Romanov family. The items are part of a collection owned by Mr. Nikolai Bachmakov, an expert restorer in Fabergé collectibles. The first item was a pre-1899 locket purchased by Mr. Bachmakov from another dealer. The locket, a Karl Fabergé pendant, was decorated with three precious stones (Figure 1A). Inside and under glass, was an old black and white photograph of Empress Alexandra Feodorovna Romanov (Figure 1B). On the other side, also under glass, was a lock of light-colored hairs. 

**Figure 2:**
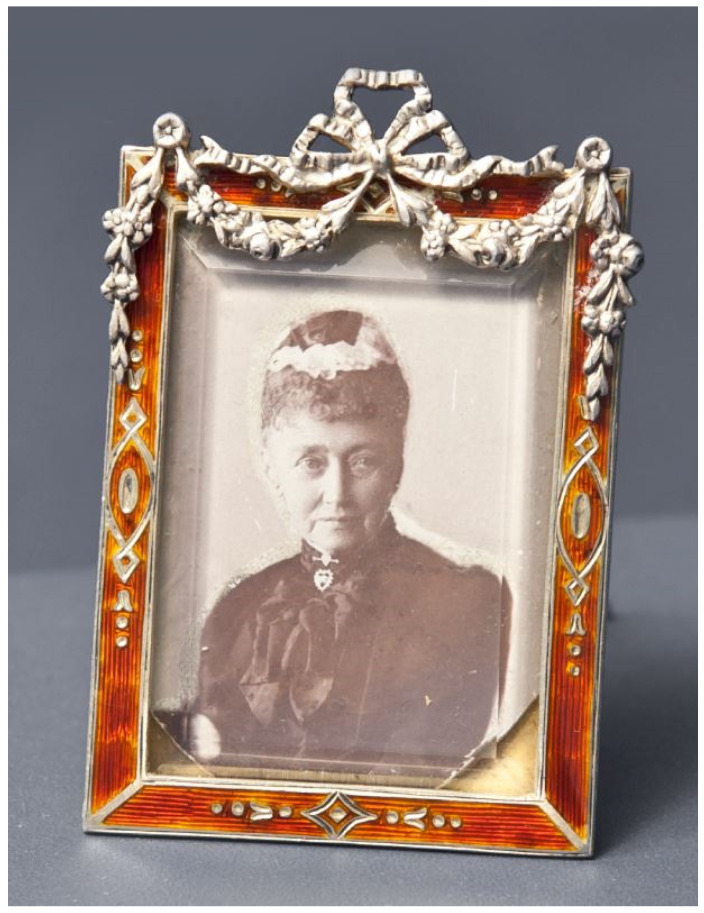
Picture frame containing a picture of Queen Louise of Hesse-Kassel after restoration. Courtesy of M. Perekrestov, from the Russian History Foundation.

Shortly after, Mr. Bachmakov informed the DSU that he was also in possession of a framed photograph of Queen Louise of Hesse-Kassel (1817–1898), wife of King Christian IX of Denmark and maternal grandmother of Tsar Nicholas II (Figure 2). Positioned between the glass of the frame and the photograph was a large lock of light hairs that were presumed to belong to Queen Louise. 

Although genetic characterization seemed to be the only way to confirm the provenance of the hairs, it was well understood that DNA recovery from such aged and degraded hairs would be challenging. MtDNA, which is sometimes targeted in forensic casework, is increasingly difficult to recover with increasing sample age. In 2005, Melton et al. [7] showed that the likelihood of obtaining a full hypervariable region I/hypervariable region II (HVI/HVII) mtDNA profile decreased from 90% in single shed hairs 0–5 years old to ~60% in hairs greater than 20 years old. Similarly, in a 2017 study, mtDNA averaged only 61 bp in human hairs that had been collected 50 to 100 years ago [8]. For older samples, next generation sequencing (NGS) techniques that do not rely on predefined amplicons have proven necessary for DNA recovery [9,10,11]. In these particular cases, it is likely that the quantities of hair that were used (between 20 mg and 2 g), the beneficial conditions of preservation, and/or the presence of some hair roots contributed to successful DNA typing.

We recently employed NGS to develop DNA data from the types of hair samples that are routinely encountered in forensic casework: single shed hairs that are generally comprised of less than 1 mg of biological material [12]. Here, we extended this work by testing even older (i.e., more degraded) rootless hairs that were believed to be associated with Russian royalty. We first assessed the feasibility of recovering DNA from hairs believed to be over a century old. Multiple hairs were pooled together for DNA extraction to improve the likelihood of DNA recovery and to optimize DNA recovery methodologies. We then applied these optimized protocols to the analysis of the types of single hair shafts that are routinely encountered in forensic DNA casework. Overall, the results not only shed light on the hypothesis that the hairs may have originated from members of the Russian Royal family, but also demonstrate the potential value of low coverage sequence data in resolving investigative questions from single decades-old-shed hairs.

## 2. Material

### 2.1. The Fabergé Locket

The Fabergé pendant is shown in Figure 1A, B. It is a 14 karat yellow gold heart locket that weighs 8.7 g and measures 24.75 mm × 19.84 mm × 7.15 mm. On the front side, a floral pattern is entwined over the heart and is embellished with three stones: one old-cut round diamond stone of ~0.10 carat (ct), one Cabochon-shaped ruby stone of ~0.12 ct, and a small rose-cut diamond of 0.001 ct (Figure 1A). 

On the back, the Russian monogram AѲ (Ѳ being an old Cyrillic equivalent to the letter F) is engraved as well as the great Imperial crown of Russia (Supplementary Figure S1C). The initials of the locket’s maker, stamped on the loose jump ring, are KΦ, as in Karl Fabergé (Figure 1B). The interior of the locket is fitted with two oval-shaped glazed picture frames and on the right side, the scratched inventory number 15,650 means that the locket was made between late 1897 and early 1898. One side contained a black and white portrait of the Empress Alexandra Feodorovna (1872–1918) and on the other side was a lock of light brown/dark blond hairs (Supplementary Figure S1A–E). 

### 2.2. The Picture Frame

The frame, made of silver 925 standard, is covered with red enamel, and decorated with silver ribbons. The size of the black and white cabinet card of Queen Louise is 6.7 cm by 4.3 cm (Figure 2). 

A picture of the pre-restored frame containing the lock of hair can be found in Supplementary Figure S2A.

### 2.3. Hair Samples

The hair samples tested from both the locket and the frame are described in Table 1. In addition to DNA testing, three hairs from the locket and one hair from the frame were examined microscopically by an FBI trace analyst examiner. These hairs were mounted on slides and permanently stored in the FBI hair reference collection.

To maximize the chances of DNA recovery, initial experiments were performed with multiple hairs. From the locket, and under the assumption that all the hairs shared the same mtDNA lineage, five hairs totaling ~16 cm were extracted (Locket 5 hairs: Lo5h). Similarly for the frame, six hairs totaling ~27 cm were tested (Queen 6 hairs: Q6h). Following extraction protocol optimization involving the replacement of MinElute columns with silica coated magnetic beads, and successful results when co-extracting multiple hairs at once, DNA extraction from single hair shafts was attempted for both historical items.

## 3. Methods

### 3.1. Microscopic Examination 

A total of four hairs were prepared on glass microscope slides using Permount mounting media (Table 1; hairs Lo1a, Lo1b, Lo1c, and Q3). The hairs were examined using a high magnification transmitted light microscope (Leica DM4000B with a Leica FS4000 optical bridge) and photographed with a Leica MC190 HD.

### 3.2. DNA Extraction Protocols

DNA extraction and library preparation were performed in a laboratory dedicated to low-quantity and low-quality DNA samples with limited personnel access. Personal protective equipment to minimize the risk of contamination included Tyvek^®^ IsoClean^®^ single-use garments (frocks, sleeves, masks from DuPont^TM^, Wilmington, DE, USA), goggles, and double gloving. The reagents were aliquoted and irradiated before the samples were introduced and all plastic consumables were irradiated in a cross-linker prior to use.

A total of three DNA extracts were generated from both the locket hairs and the frame hairs, for a total of six extracts. Of these six extracts, two were developed from multiple hairs (Lo5h and Q6h), and four were developed from single hair shafts (Lo2, Lo3, Q1, and Q2).

Two different purification methods were used. Protocol B from Brandhagen et al. [12] was applied first for the extractions of multiple hairs samples (Lo5h and Q6h). In brief, DNA was extracted using a lysis buffer optimized for hair digestion and then purified with MinElute silica columns from the Qiagen PCR purification kit (Qiagen, Germantown, MD, USA) using large volumes of binding buffer. To accommodate the large volumes of solution, the MinElute columns were fitted with Qiagen extension columns and placed on a vacuum manifold (VacConnector, Qiagen).

DNA from single strands of hair was extracted using the same optimized digestion buffer and volumes as in protocol B, but instead of using MinElute columns, DNA was purified with magnetic beads coated with silica (cat#: 786–915, G-Bioscience, Maryland Heights, MO, USA) as recommended by Rohland et al. [13]. A detailed description of the new protocol can be found in Supplementary Material #1.

DNA quantification via qPCR was not performed on the extracts, as previous experiments had demonstrated that DNA fragments present in hair samples of considerable age are generally too small to yield a 105 bp amplicon with the mtDNA qPCR assay routinely used in DSU [12,14]. Although fluorometric quantification was attempted using a Qubit^TM^ Fluorometer and a dsDNA High Sensitivity Assay Kit (Invitrogen, Waltham, MA, USA), no dsDNA could be detected. The final concentration of the adapters was arbitrarily set at 300 nM.

### 3.3. Library Preparation

A total of 50 μL from all six DNA extracts (Lo5h, Lo2, Lo3, Q6h, Q1, Q2) and four reagent controls (RB1 to RB4) were converted into double stranded DNA Illumina libraries using the NEBNext^®^ Ultra^TM^ II DNA Library Prep Kit for Illumina^®^ (NEB, Ipswich, MA, USA) and looped adapters from the NEBNext^®^ Multiplex Oligos for Illumina^®^ kit (NEB). Illumina libraries were prepared according to the manufacturer’s instructions except for ligation which used highly diluted adapters and was performed overnight at 7 °C. Following ligation, the ligated looped adapters were converted into Y-shaped adapters with the USER^®^ Enzyme kit (NEB). The libraries were then purified with 1.8× AMPure XP beads (Beckman Coulter, Sykesville, MD, USA). All the libraries were dual indexed with primers from the NEBNext^®^ Multiplex Oligos for Illumina^®^ kit and subsequently amplified for 25 cycles with the NEBNext^®^ Q5^®^ Hot Start HiFi PCR Master Mix (NEB). Purification of the amplified libraries was performed using 1.5× AMPure XP beads. The libraries were then shotgun sequenced on an MiSeq Forensic Genomics System (FGx^TM^) instrument (Illumina Inc., San Diego, CA, USA) with a v2 cartridge and 2 × 151 cycles and/or on a NextSeq 500/550 (Illumina) with a High Output kit v2.5 and 2 × 75 cycles in order to obtain additional data.

### 3.4. Hybridization Capture

Mitochondrial DNA enrichment via hybridization capture was performed on all RBs and on four libraries, one from the locket (Lo5h) and three from the frame (Q6h, Q1, and Q2), using biotinylated RNA baits (myBaits Mito Human, Modern Global, Daicel Arbor Biosciences, Ann Arbor, MI, USA). Each hybridization capture was performed at 62 °C for 24 h using 100 ng of 80 bp long RNA baits. The captured product was then amplified for 16 to 19 cycles and purified with AMPure XP beads. DNA from the purified enriched libraries was successfully quantified using a Qubit^TM^ Fluorometer and the Qubit^TM^ dsDNA Broad Range Assay Kit (Invitrogen), then diluted and analyzed on a 2100 Bioanalyzer with the High Sensitivity DNA Kit (Agilent, Santa Clara, CA, USA). Following quantification, libraries were pooled and sequenced on a MiSeq FGx^TM^ instrument with a v2 cartridge and 2 × 151 cycles. The very first capture library that was sequenced, Lo5h-mt, was also sequenced on the NextSeq 500 with 2 × 75 cycles. The comparison of the data from both instruments showed that a MiSeq run was sufficient to accurately sequence the small mtGenome. As a result, all the subsequent capture libraries were only sequenced on the MiSeq FGx^TM^.

### 3.5. Data Analyses

Raw Fastq reads were first imported as paired-end reads, quality trimmed, and the overlapping pairs were merged in the CLC Genomics Workbench, version 12.0 (CLC Bio/Qiagen, Aarhus, Denmark). Merged reads were then mapped to the human genome by a supercomputer using the Burrows-Wheeler Aligner (BWA aln, v0.7.17–r1188) with seed disabled [15] and SAMTools v.1.11 [16]. BWA aln was chosen over CLC because this algorithm is more efficient at mapping very short reads (i.e., reads under 50 bp). Reads with mapping quality below 30 were removed and, to circumvent the fact that BWA can’t map reads to a circular reference, a chimeric mtDNA reference was created to allow reads that overlap the mtGenome origin to map. The 33,152 bp sequence was developed by combining the sequence of the standard revised Cambridge reference (rCRS) 16,569 bp genome, followed by 14 N and a second mtGenome sequence starting at position 8284 and ending at position 8283. This chimeric mtDNA reference also replaced the mtDNA reference ChrM in the human genome reference hg19 (a.k.a GRCh37). Duplicate reads were removed using Picard v.2.23.8 [17]. Mappings were imported back into CLC as bam files and the mtDNA reads were extracted and re-mapped to the standard 16,569 bp circular rCRS reference. Duplicate mtDNA reads were removed and CLC was used to develop variant calls, DNA size distribution, and read depth. The shotgun data from single hair extracts were used to assess biological sex using the R_X_ method published by Mittnik et al. [18]. This method compares the number of sequences originating from the X chromosome to the number of sequences originating from the 22 autosomes. A male assignment is made if the upper bound of the 95% CI is less than 0.60, while a female assignment is made if the lower bound is greater than 0.80.

**Figure 3:**
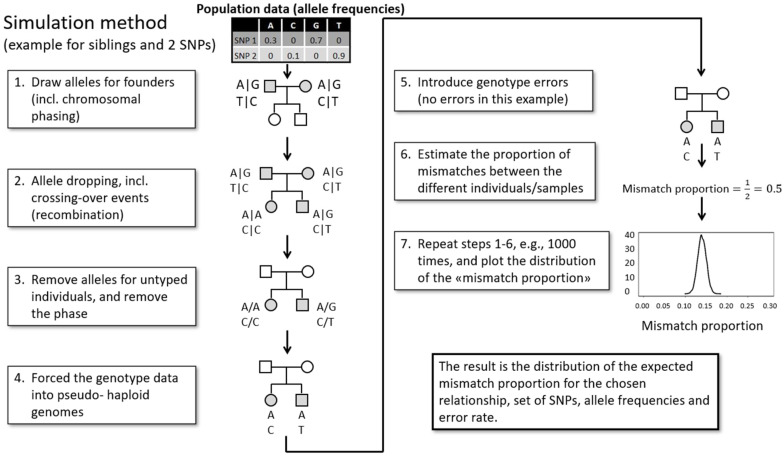
Simulation approach applied to infer the degree of relatedness between low coverage DNA datasets based on allele mismatch proportions.

Next, the shotgun data were used to analyze ~1.3 million nuclear SNPs that were covered by at least one read. From the SNP results, a “forced haploid” approach was used to assess relatedness between the two locket hairs, Lo2 and Lo3 (Figure 3).

In brief, relatedness was assessed by comparing allele mismatch proportions between the two locket hairs to expected allele mismatch proportions for unrelated, parent-child, sibling, and same individual relationships. Specifically, the shotgun datasets from Lo2 and Lo3 were first reduced to 1.3 million SNPs that include the SNPs generally typed by genetic genealogy companies and used in GEDmatch [19,20]. The number of SNPs was then further reduced to SNPs that overlapped between the two hair samples. Next, using a custom R script, the datasets were forced into pseudo-haploid genomes for which the allele mismatch proportion was calculated. The resulting SNP marker set (i.e., SNPs with allele calls in both samples) was used as the input in the mismatch proportion simulation method. To obtain expected mismatch proportion distributions, pseudo-haploid datasets for various degrees of relatedness (i.e., same individual, parent/child, full siblings, or unrelated individuals) were simulated using the European allele frequencies of the Genomes Project Consortium [21] and SNP-nexus [22,23] for the specific set of SNPs that were included in the sample comparison. In addition, a simplistic error model was included to account for the possibility of different genotype errors such as deamination, depurination, PCR error, sequencing error, and mapping error, that may occur for low-quality and low-quantity DNA samples. An error rate of 0 to 2% was used in our simulations. As controls, shotgun data from two 50 year-old-sourced hair samples, completely unconnected to this project and that were known to originate from the same individual (189A and 189B) were analyzed and compared. In addition, data from hair samples 189A and Lo2, which were known to originate from two unrelated individuals, were compared (a more detailed description of the method can be found in Supplementary Material #2). 

Finally, some of the unmapped reads were analyzed using the Metagenomics RAST (MG-RAST) server [24] in an effort to identify the source of the non-human reads.

## 4. Results

### 4.1. Histology Examination

**Figure 4:**
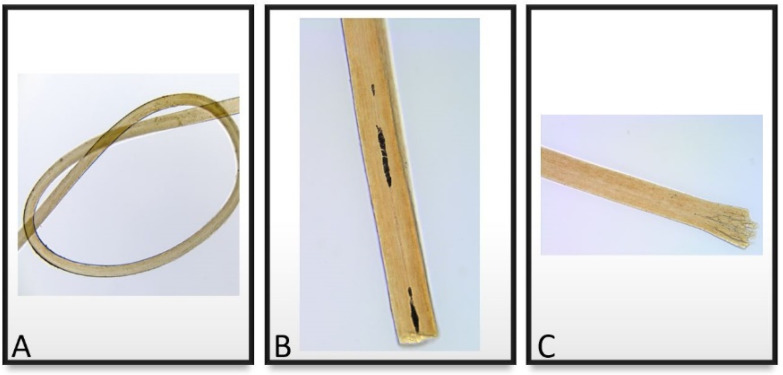
Photographs taken from three different hairs that were found in the Fabergé locket. (**A**) Magnification 100×. (**B**) Magnification 200× shows that the proximal end of the hair appears cut. (**C**) Magnification 200× shows that the proximal end of the hair appears broken.

**Figure 5:**
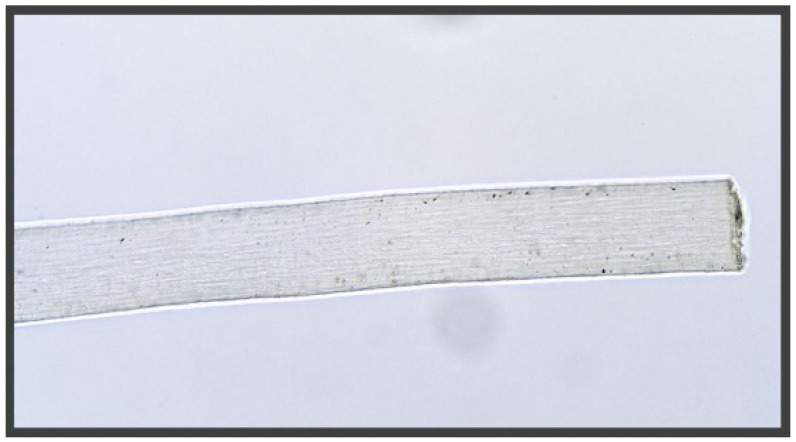
Magnification: 200×. Photograph taken from a white hair that was found in the picture frame. The proximal end of the hair appears cut.

The four microscopically-examined hairs were very well preserved overall (Figure 4A–C and Figure 5) as characteristics of decomposition, insect damage, or fungal damage were not observed. Nor was there any indication of artificial treatment, such as dyeing or bleaching. Based on pigment arrangement, pigment density, overall diameter, cross-sectional shape of the hair shaft and the cuticle thickness, the examiner determined that all four hairs were most likely human head hairs of European ancestry. 

Based on microscopic examination, it was established that no root was present on any of the samples examined.

### 4.2. Illumina Sequencing Results from the Fabergé Locket Hairs

Three libraries were produced with the locket samples. From sample Lo5h (extract/library from five hairs), a shotgun DNA library (Lo5h-sh, a library not enriched for any marker) as well as a mtDNA-enriched library (Lo5h-mt) were sequenced on a MiSeq FGx^TM^. The mtDNA-enriched library Lo5h-mt was also sequenced on a NextSeq 500 to compare the data that were obtained from both instruments. For single hair extracts/library Lo2 and Lo3, shotgun libraries were only sequenced on a NextSeq 500 and no hybridization capture was performed. Sequencing statistics for the hair samples that were found in the locket are presented in Table 2 and more detailed information can be found in Supplementary Table S1. Shotgun library Lo5h-sh was only sequenced on a MiSeq FGx^TM^ since our focus for the multi-hair extracts was to establish that DNA could be recovered from hairs of this age, and that is best addressed by targeting the mtGenome. In addition, since the locket had a picture of Tsarina Alexandra, it was conceivable that it contained hairs from herself and/or from some of her children. As a result, all the individuals would share the same mtGenome and data analysis would not be compromised by mixtures. Analysis of nuclear DNA, however, would have presented a challenge. Consequently, high throughput sequencing of Lo5h-sh for nuclear DNA analysis was not attempted on the NextSeq 500 platform.

The percentage of endogenous human reads in the Lo5h-sh library was 48%. This percentage was a bit lower than expected considering that all hairs had been thoroughly decontaminated via Terg-a-zyme sonication before digestion. When unmapped reads of 75 bp or longer were analyzed with MG-RAST, close to 63% of the sequences remained unknown and most of the sequences that matched a sequence in GenBank belonged to the Proteobacteria phylum (Supplementary Material #2).

The percentage of endogenous human DNA was, however, higher when single hairs were extracted with the optimized protocol (66.54% for Lo2-sh and 78.91% for Lo3-sh). Additionally, as a precaution, purification reagents remained stored at 4°C between use to avoid potential mold growth. In this case, since both Lo2 and Lo3 were single source samples, high throughput sequencing of the libraries was carried out on the NextSeq 500 platform to collect as many DNA sequences as possible.

#### 4.2.1. Mitochondrial DNA Results

**Figure 6:**
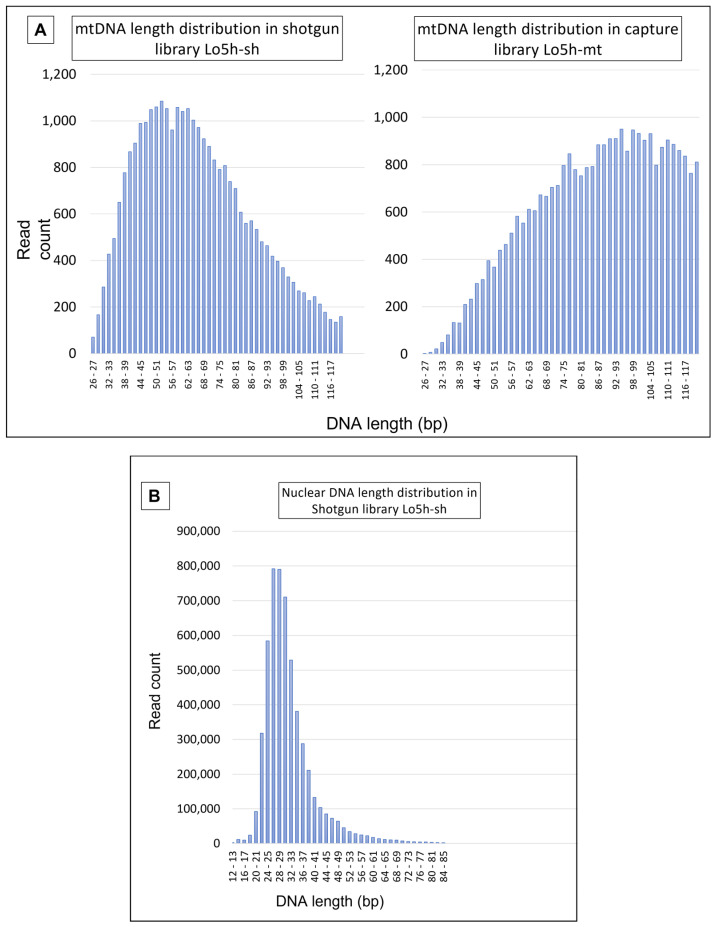
Length distribution of (**A**) mtDNA reads in Lo5h-sh and Lo5h-mt, and (**B**) nuclear DNA reads in Lo5h-sh.

MtDNA-enriched library Lo5h-mt produced 35,188 unique mtDNA reads representing >3.2 million bases. The entire mtGenome was successfully sequenced at an average depth of 198× (range: 159–273×; standard deviation (SD): 11.38). Shotgun sequencing of the Lo5h-sh produced 29,879 mtDNA reads representing >1.9 million bases. Once again, the complete mtGenome was sequenced at an average depth of 119× (range: 5–173×; SD: 17.77). The DNA length distributions of Lo5h-sh and Lo5h-mt are presented in Figure 6A.

Shotgun libraries Lo2-sh and Lo3-sh yielded 27,058 and 32,099 unique mtDNA sequences with an average depth of 111× (range: 22–148×; SD:13.08) and 164× (range: 26–219×; SD:12.06) respectively. MtDNA enrichment of the Lo2 and Lo3 libraries via hybridization capture was deemed unnecessary since there was sufficient coverage of the mtGenome in the shotgun data that were produced by the NextSeq.

The average mtDNA fragment length varied between 66.04 bp and 93.46 bp, depending on the library and the reference used for mapping (Table 3 and Table S5). Interestingly, the hybridization capture library that was developed from Lo5h (Lo5h-mt) yielded an average mtDNA fragment size that was much larger than the one that was observed in the Lo5h shotgun library (Lo5h-sh). 

At 93.46 bp, the mean length of the mtDNA reads in the enriched Lo5h-mt library was substantially higher than the mean mtDNA length that were observed in the shotgun libraries (Table 3). This pattern between the shotgun and enriched libraries has been observed in several other studies involving ancient DNA [25,26,27]. Our hypothesis that the noticeable disparity in average length could be caused by a preferential binding of larger mtDNA molecules during hybridization capture was confirmed by Krasnenko et al. [28] who stated that “[…] insert size crucially impact(s) enrichment results” and that “the maximum efficiency of enrichment (>90%) is achieved with 250–330 bp insertion length”. Of note, average DNA length estimates should be viewed with the following caveats in mind: they can be greatly influenced by factors such as DNA extraction protocol, library preparation protocol, number of sequencing cycles, and bioinformatics pipelines (trimming in particular).

Shotgun libraries Lo2-sh and Lo3-sh also showed substantial variation in mean mtDNA fragment length (67.82 versus 84.99 bp). These differences could be the result of a number of factors, including sample variability between individual hairs or variability resulting from the distance between the tested section and the root [12]. 

All the locket hair libraries produced the same mtGenome profile: 263G, 315.1C, 524.1A, 524.2C, 750G, 1438G, 3010A, 4137T, 4769G, 8860G, 15326G, 16111T, 16357C, and 16519C. The sequence belongs to haplogroup H1af2 and is identical to the mtGenome of Empress Alexandra’s maternal lineage published as “Queen Victoria’s profile” by Rogaev et al. [6], (GenBank # FJ656214). Detailed variant tables can be found in Supplementary Table S2.

#### 4.2.2. Nuclear DNA Results

Sequencing of the shotgun libraries produced 38,008,962 (Lo2-sh), 33,328,275 (Lo3-sh), and 5,470,833 (Lo5h-sh) unique human nuclear sequences representing 1,256,322,853 (Lo2-sh), 1,104,095,334 (Lo3-sh), and 174,843,798 (Lo5h-sh) nucleotides, respectively. The average read depth was 0.40× (Lo2-sh), 0.35× (Lo3-sh) and 0.06× (Lo5h-sh), and the average nuclear DNA fragment length was 33.05 (Lo2-sh), 33.13 (Lo3-sh) and 31.96 bp (Lo5h-sh) (Table 4). In every library, at least 98.9% of the human DNA that was sequenced belonged to the nuclear genome.

As has been previously observed in Brandhagen et al. [12], the average length of nuclear DNA fragments tends to be smaller than the average length of mtDNA fragments. 

#### 4.2.3. Biological Sex

The two single hairs that were shotgun sequenced (Lo2-sh and Lo3-sh) were assessed for biological sex. In both cases, the lower bound of the Rx confidence interval was >0.8, resulting in a biological sex of female (Table 5). This result excludes the Romanov’s son, Tsarevich Alexis, as a possible source of these two hairs.

#### 4.2.4. Relatedness Estimates

**Figure 7:**
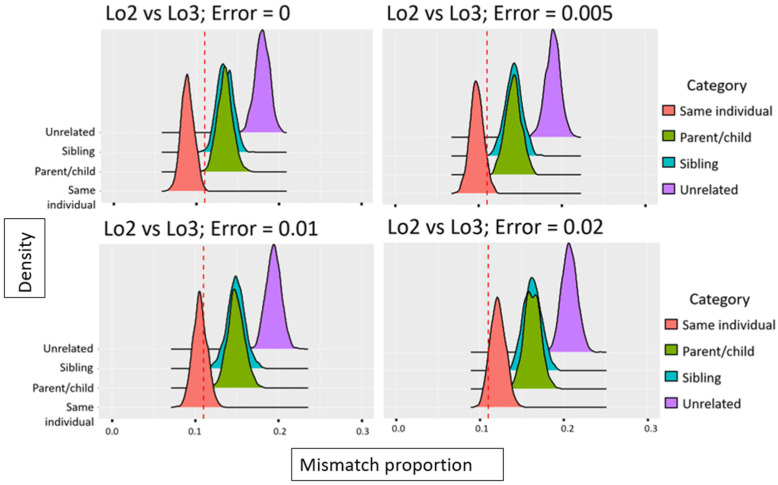
Observed mismatch proportion for the Lo2 vs. Lo3 comparison (red dashed line) in relation to the expected mismatch proportions for various degrees of relatedness and different genotype error rates.

In addition to being assessed for biological sex, hairs Lo2 and Lo3 were tested for relatedness. Only reads >35 bp were used for this analysis to avoid spurious mapping of bacterial sequences, as recommended by Meyer et al. [29]. Out of 1.3 million SNPs, 15,331 and 33,591 were covered by at least one read for Lo2-sh and Lo3-sh, respectively, and 1196 of these SNPs were covered in both samples. The proportion of allele mismatches was calculated to 0.11 after forcing the Lo2-sh and Lo3-sh datasets into pseudo-haploid genomes. Assuming a non-zero genotype error rate, such a proportion is expected among samples originating from the same individual and is, at the same time, unexpected for parent/child, siblings, and unrelated individuals (Figure 7). As controls for the forced haploid simulation approach, the mismatch proportions for the two known samples originating from the same individual as well as two samples originating from two known unrelated individuals were determined. The observed mismatch proportions for those comparisons fit well with the expected mismatch distributions for same individual and unrelated individuals, respectively (Supplementary Material #2). Based on the observed forced haploid mismatch proportion as evaluated against the simulated data and considering the error rate that is most appropriate for the given comparison, the data indicate that Lo2 and Lo3 originate from the same individual. 

### 4.3. Illumina Sequencing Results from the Picture Frame Hairs

Sequencing statistics for the hair samples that were found in the frame are shown in Table 6. The low percentage of endogenous human DNA in the library derived from six hairs (Q6h-sh) (21.17%) is even lower than the 48% that was observed with the library from five locket hairs (Lo5h-sh). However, as with the locket hairs, the percentage of human DNA improved substantially in the libraries that were derived from single hair shafts. Sample Q1-sh produced 70.12% human DNA, while Q2-sh produced 89%. The three hybridization capture libraries, not surprisingly, yielded the highest percentages of target DNA. In all three cases, ≥88% of the human reads were mtDNA sequences. Further details can be found in Supplementary Table S1.

Among the shotgun libraries, the number of endogenous molecules in Q1-sh was noticeably lower than in the other samples (Table 6). The high quantity of damage in this sample, exacerbated by an over- amplification of the DNA, resulted in a high percentage of noise in the data. As a result, the Q1-sh library was not sequenced on the NextSeq 500 instrument and fewer unique human reads were, therefore, recovered.

#### 4.3.1. Mitochondrial DNA Results

Since the frame hairs seemed more degraded than the locket hairs, every Q library was enriched for mtDNA to produce the highest possible data quality. 

For Q6h-mt, 26,038 unique mtDNA reads were obtained (>1.2 million bp) and the entire mtGenome was sequenced at an average depth of 74× (range: 27–117×; SD:11.45). At 47.26 bp, the average length of the DNA fragments was significantly shorter than the 93.08 bp average mtDNA size that was observed with Lo5h-mt. 

For Q1-mt, a total of 14,514 unique mtDNA reads, spanning 563,192 bp were sequenced. In this case, the average read depth was 34× (range: 1–100×; SD: 14.98) and the average mtDNA length was 38.80 bp. 

Finally, for Q2-mt, 24,636 unique reads totaling >1.1 million bp were recovered. Reads spanned the entire mtGenome, with an average read depth of 67× (range: 13–125×; SD: 15.49) and an average fragment length of 45.33 bp.

Mitochondrial DNA was also analyzed from the shotgun sequence data of Q6h-sh and Q2-sh (Table 7). 

**Figure 8:**
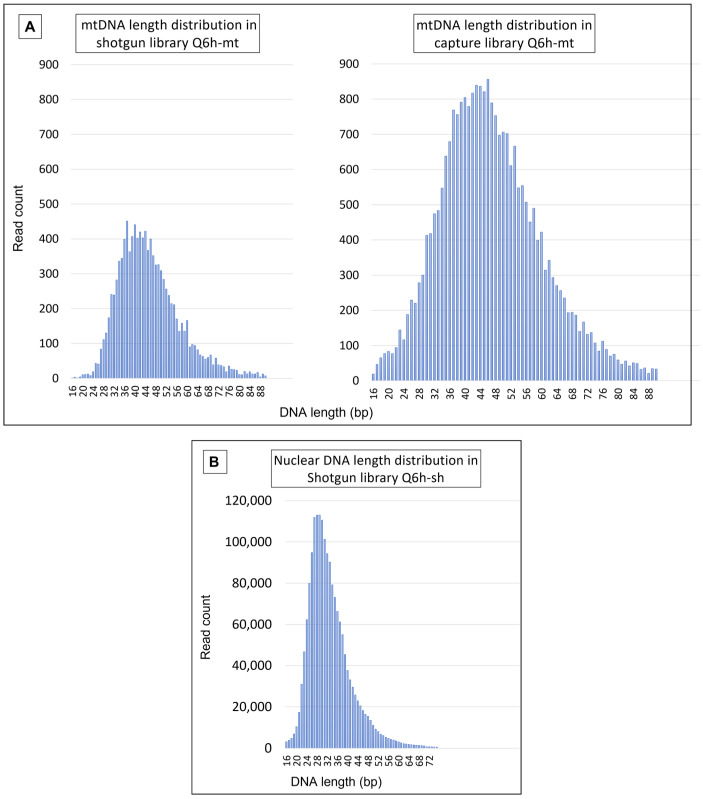
Length distribution of (**A**) mtDNA reads in Q6h-sh and Q6h-mt, and (**B**) nuclear DNA reads in Q6h-sh.

The 1281 mtDNA reads obtained with Q1-sh were insufficient to produce reliable variant calls. Similar to what was observed with the locket hairs, the average mtDNA fragment size in the capture libraries was larger, although the magnitude of the difference was relatively small (Figure 8). This is likely due to the fact that the range of the average lengths for the mtDNA fragments in the frame hairs was smaller than the range that was observed with the locket hairs.

Capture library Q6h-mt and shotgun library Q6h-sh both produced the following mtDNA profile: 73G, 263G, 315.1C, 709A, 750G, 1438G, 1842G, 1888A, 2706G, 2850C, 4216C, 4769G, 4917G, 6257A, 7022C, 7028T, 8697A, 8860G, 10463C, 11251G, 11719A, 11812G, 13368A, 13965C, 14233G, 14687G, 14766T, 14905A, 15326G, 15452A, 15607G, 15928A, 16126C, 16169Y, 16294T, 16296T, 16519C. The profile belongs to haplogroup T2a1a and is identical to the published sequence of Tsar Nicholas Romanov, including position 16169 which is characterized by a point heteroplasmy in the Tsar and his brother’s mtGenome [3,4,5,6], (GenBank # FJ656215). Position 16169 had a read depth of 75× with 57 Ts and 18 Cs (76% T). In the shotgun data, the percentage of T versus C varies between 80% and 81% depending on the reference used for mapping. The reference can cause bias as a result of pseudogenes in the human genome. If data are mapped against hg19, which includes both the nuclear and the mitochondrial genome, unspecific sequences will be randomly mapped to one genome or the other, and true mtDNA sequences may be erroneously mapped to nuclear pseudogenes. On the other hand, if the data are mapped only to the rCRS, pseudogene sequences (although rare in these types of degraded samples) could erroneously map to the mtGenome. For this reason, the shotgun data were mapped to both references.

Since the Q6h library was prepared using six hairs, it is not possible to determine whether individual hairs were heteroplasmic or homoplasmic. That is, the mixed position at 16169 in both the shotgun and capture libraries could be the result of individual hairs being homoplasmic for the two variants.

The mtGenome profile that was produced by Q1-mt is similar to the Q6h-mt profile with one exception: position 16169 appeared homoplasmic for thymine, with 27 out of 27 reads exhibiting a T.

In contrast to Q1-mt, position 16169 in library Q2-mt reflected primarily cytosine bases, with 53 C and 1 T. Since hydrolytic deamination is one of the most abundant forms of damage in ancient molecules, it is possible that the thymine base that was detected in the data was a deaminated cytosine [30,31]. In fact, in the shotgun library, all 25 reads have a C which supports 16169 being homoplasmic for cytosine.

#### 4.3.2. Nuclear DNA Results 

Sequencing of the library Q6h-sh produced 1,806,916 nuclear reads representing close to 60 million bases, an average read depth of 0.02× (range: 1–109×; SD: 1.04), and a mean nuclear DNA length of 33.18 bp. Sequencing of library Q1-sh on a MiSeq FGx^TM^ produced 238,411 unique human nuclear sequences for a total of 8.4 million bases. The average read depth was <0.01× and the mean DNA length was 35.51 bp. 

Finally, sequencing of library Q2-sh on a NextSeq 500 platform produced 56,375,439 unique human nuclear reads representing a total of 1.8 billion nucleotides. The average read depth was 0.58× (range: 1–184; SD: 4.65) and the median nuclear read length was 32.06 bp. 

Unfortunately, the number of quality reads that were recovered from Q1-sh was insufficient to perform a relatedness analysis between Q1 and Q2. However, the biological sex could be assessed.

For both hairs, the lower bound of the confidence interval was >0.8 (Table 8), resulting in a biological sex of female, the expected result if the hairs belonged to Queen Louise.

## 5. Discussion

The primary goal of this study was to explore the origin of the hairs in the locket and the frame using techniques and, more importantly, sample sizes that are relevant to forensic casework. By using these historical samples as a proxy for evidentiary material, protocols for extremely limited and compromised samples could be further optimized for routine casework. Overall, the results demonstrate that this method can successfully recover both full mitochondrial genomes and nuclear DNA data from the types of single, aged, and degraded hair samples that are routinely encountered in forensic casework. Furthermore, the results illustrate that limited genetic information can be useful for answering questions that are relevant to DNA identification. Specifically, the comparison between the single strands of hair that were tested from the locket indicate that despite partial SNP information for any given hair sample and limited overlapping data between any two hair samples, valuable information can be acquired. Typical forensic samples rarely, if ever, meet or exceed the suspected age and degradation states of the hairs tested here, yet the integrity of DNA in older cases with hairs recovered from less optimal storage conditions or that were originally collected from harsher environmental settings than those that were encountered here may make these studies directly applicable to some casework scenarios. Hence, the development of protocols for samples that challenge or exceed the lower detection limits of the currently employed methods demonstrates potential for the utility of the protocols in even the worst, or at least the vast majority of casework scenarios.

### 5.1. DNA Extraction Modifications

To maximize DNA recovery from the hair shafts, modifications were made to the purification step of the DNA extraction. Previous work has shown that both mtDNA and nuclear DNA can be obtained from single shed hairs up to 60 years old with a DNA extraction protocol that employed the MinElute PCR purification kit [12]. This particular protocol/purification method was used as a starting point to assess the feasibility of recovering DNA from hair this old, and extractions were performed with five (Lo5h) and six (Q6h) hairs to maximize the chances of detecting DNA. 

**Figure 9:**
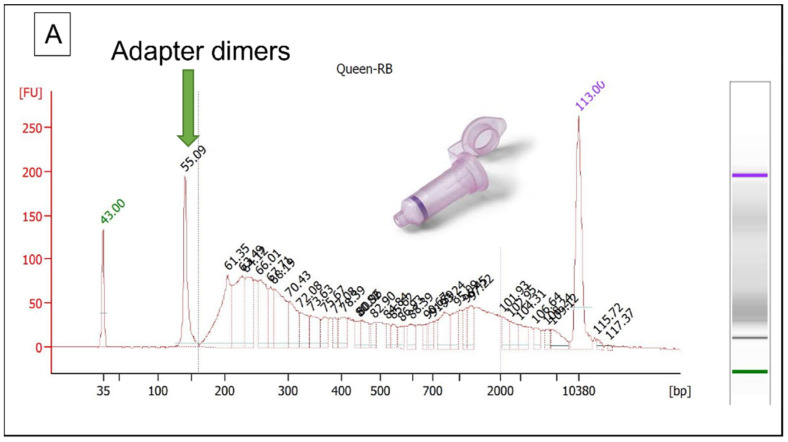

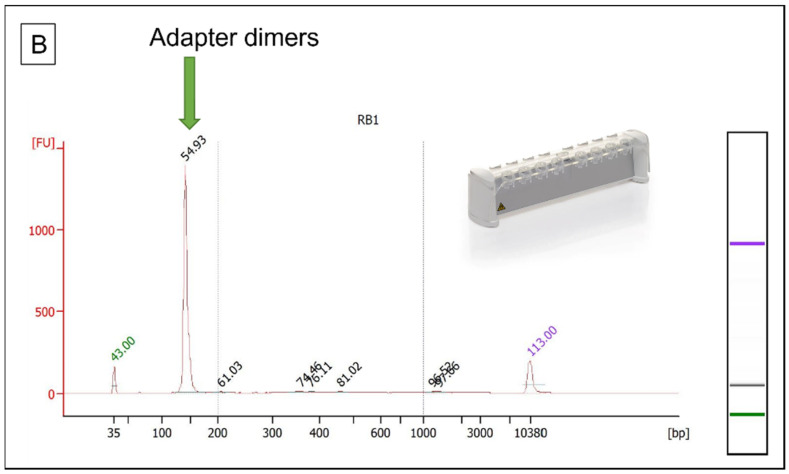
Bioanalyzer electropherograms of reagent blank libraries when DNA purification was performed using (**A**) MinElute columns, or (**B**) magnetic beads from G-Biosciences.

When these two libraries were evaluated with the 2100 Bioanalyzer, the electropherograms of the reagent blanks showed a large curve that was similar to the ones that were observed with the hair libraries (Figure 9A). 

The sequencing of these reagent blank libraries produced data but the reads did not map to the human genome reference. These results suggest that the MinElute columns are contaminated with trace amounts of non-human DNA. For most human DNA identification applications, this type of very low-level contamination would have little impact on the final sequence data. Given that the contaminant is non-human and present only in trace quantities, the ratio of target human DNA to contaminant DNA would be high enough for the contaminant to go undetected. However, when the number of PCR cycles is high and when only a few target molecules are present in the extract, such as in the aged and degraded single hair shaft extracts that were tested here, the ratio of endogenous DNA to non-human contaminant DNA drops substantially. The MinElute columns were, therefore, replaced first with the Ultraclean Production (UCP) MinElute columns (cat # 56204, Qiagen). Though Bioanalyzer profiles of hair libraries that were purified with the UCP columns did not reflect the presence of any DNA, the columns were not used on the samples that were presented in this study for two reasons: (1) the use of a vacuum and extender columns was cumbersome and contamination-prone and (2) use of the columns resulted in a near doubling of library purification costs from ~$6 USD per sample to over $11 USD per sample. The columns were thus replaced with silica beads from G-Biosciences. These beads produced the same library purity as the UCP columns, but at a cost of ~$5 USD per sample. In addition, experiments that were performed on other aged hairs showed that the average size of DNA molecules that were collected by silica beads was slightly smaller than the DNA that was purified with MinElute and UCP MinElute columns. As a result, the columns were replaced by the more practical and cost-efficient DNA-free silica magnetic beads for all subsequent experiments.

The sequencing results of the bead-purified hair extracts demonstrated a substantial increase in the percentage of human DNA that was recovered (Table 2 and Table 6). For the locket hairs, the recovery of human DNA improved from 48% in the MinElute-purified library (Lo5h-sh) to 66.54% (Lo2-sh) and 78.91% (Lo3-sh) in the two bead-purified shotgun libraries. Similarly, for the frame hairs, human DNA recovery increased from 21.17% (Q6h-sh) to 70.12% and 89% in Q1-sh and Q2-sh, respectively. Finally, unlike the Bioanalyzer electropherograms from the MinElute-purified libraries, the bead-purified RB library electropherograms exhibited only a single 140 bp peak of adapter dimers (Figure 9B).

In Brandhagen et al. [12], the previous version of our protocol had been tested as a way to save ultrashort DNA fragments that were lost during a wash step from the PrepFiler Kit protocol (Applied Biosystems, Waltham, MA, USA). We confirmed that the optimized silica beads protocol works equally well, or better, than the previous one in small fragment retention. 

### 5.2. Authenticity of the Items

DNA testing is often employed to authenticate items of historical significance [32]. However, successful DNA testing is dictated by the type of item, its age and condition, how it has been stored, whether it has been handled/contaminated, etc. Items that can be decontaminated, such as hairs and calcified tissues, generally offer the best chance of success. Yet, even in cases for which the target DNA can often be recovered, authentication can still prove difficult, particularly when the direct or familial references that are required for identification are not straightforward to establish or acquire [33]. Fortunately, for historical items that are believed to be associated with the Romanov family, sufficient DNA reference information exists. Thus, on top of the historical evidence tying the locket and frame artifacts to the Romanov family, the DNA data that were recovered from the hairs may also prove useful.

#### 5.2.1. Fabergé Locket

##### Historical Evidence of Authenticity

The photograph of Princess Alexandra displayed in the locket comes from a cabinet card, a type of photography mounted on a piece of cardboard that was very popular in the 1860s. It could be confidently identified as a copy of a photograph that was made in St Petersburg in 1895 by Alexander Pasetti, the photographer of the Imperial Russian court (see the original photograph in Supplementary Figure S4). 

The pendant was crafted by the famous Russian jeweler and goldsmith, Karl Gustavovich Fabergé (also known as Peter Carl Fabergé; 1846–1920). Karl was the son of Gustav Fabergé, a German jeweler who moved to St. Petersburg in 1842 and opened the House of Fabergé. The association between Karl Fabergé and the Russian Imperial family is well documented. On Orthodox Easter of 1885, Tsar Alexander III Romanov offered his wife, Empress Maria Feodorovna, a jeweled Eastern Hen egg that was made by Fabergé [34]. The Empress liked it so much that Alexander named Fabergé goldsmith by special appointment to the Imperial crown and commissioned an Easter egg every following year. After Alexander’s death in 1894, his son, Tsar Nicholas II, took over the tradition and asked Fabergé to create two unique eggs each year, one for his mother, the Dowager Empress Maria, and one for his wife Alexandra Feodorovna [35]. While Easter eggs made the House of Fabergé famous, they only represent a fraction of the Fabergé creations that were made for the Tsars and their families [34].

##### Genetic Evidence

The Tsarina’s mtGenome was characterized in 2009 [6]. Hence, it was possible to directly compare the mtGenome haplotype that was developed from the locket hairs to the reference haplotype for the Tsarina; the two sequences are identical. The haplotype belongs to haplogroup H1af2 [36], which is characterized by a C4137T transition. To understand the rarity of the haplotype, three different mtDNA datasets were searched: the public FamilyTreeDNA© mitochondrial DNA Haplotree database [37], GenBank, and the European DNA Profiling Group mtDNA Population database (EMPOP, [38]). Sequence level data could not be compared in the FamilyTreeDNA© dataset. However, only ten of 170,000 complete mtGenome sequences were reported to belong to the H1af2 haplogroup. When the complete mtGenome sequence is compared to the GenBank database, no match is returned besides the profiles of the Tsarina, Alexei, and Maria, that were published by Rogaev et al. [6]. In fact, of more than 52,000 mtGenome profiles present in GenBank, only 38 sequences have a C4137T, and only five of those (including the three profiles mentioned above) belongs to haplogroup H [39]. H1af2 sequences are not currently present in EMPOP. While it is not possible to establish the identity based on mtDNA, the rarity of this profile increases the odds that the hairs that were found in the locket belonged to the Tsarina or a member of her maternal lineage.

To gain further insight into the source of the hairs and, in more general terms, explore the potential probative value of the nuclear DNA data that were recovered, autosomal SNPs were also analyzed. Autosomal SNP reference data was not available for the Tsarina or her relatives, so our goal with the SNP analysis was not source identity. Instead, the SNP results were used to assess if the strands of hair in the locket originated from the same individual or from multiple individuals from the same maternal lineage. Assuming the hairs belong to the Tsarina or one of her immediate maternal relatives (i.e., her children), the hairs would be over 100 years old. If this is indeed the case, the presence of mtDNA fragments that were larger than 90 bp in both the shotgun and the enriched library of Lo5h suggests that at least a portion of the molecules was well preserved. 

In previous studies, hairs of somewhat similar age have yielded average mtDNA fragment sizes of approximately 60 bp [8], while younger hairs (between 40 and 60 years old) were shown to harbor mtDNA fragments averaging 70–80 bp [12]. There could be at least two explanations for the great preservation of the locket hair DNA. First, according to the locket owner, Mr. Bachmakov, the locket was extremely difficult to open and required specialized tools to do so. In Mr. Bachmakov’s expert opinion, the locket had not been opened for years, perhaps even decades, and it is likely that the hairs remained safely sealed and protected from oxygen, light, and humidity. Second, saw dust that was found inside the locket may have acted as a desiccant. In the 19th and 20th centuries, jewels that were made of gold were treated with acid to enrich the proportion of gold on the surface and to change the color from a pink to a yellow gold [40]. After being rinsed with water, the objects were dried and saw dust was used to absorb moisture. In some cases, as in the case of this Fabergé locket, small amounts of saw dust remained inside the jewels. Unbeknown to the maker of the pendant, the desiccant properties of the saw dust may have played a key role in preserving the hairs and their genetic material.

#### 5.2.2. Picture Frame

##### Historical Evidence

The origin of the framed picture of Queen Louise of Hesse Kassel is supported by documents that were provided to Mr. Bachmakov when the object was purchased. The paperwork indicates that the framed photograph had originally belonged to Maria Feodorovna (known as Princess Dagmar of Denmark prior to her marriage), one of Louise’s daughters. It was passed down to one of Maria Feodorovna’s daughters, Grand Duchess Xenia (1875–1960), and then again through various family members to a living descendant who kept it in his/her possession until 2017 when it was purchased by Mr. Bachmakov. The picture also comes from a cabinet card and the initials on the back show that the photograph was taken by Mary Steen, a famous Danish photographer who, in 1896, was appointed court photographer to Alexandra, Princess of Wales (1844–1925), later Queen Alexandra (Supplementary Figure S5). 

Given the picture of Queen Louise and the fact that the strands of hair were white, it is presumed that they came from the Queen and were collected from her in her senior years. If these assumptions are correct, the hairs would be at least 120 years old.

##### Genetic Evidence and mtDNA Heteroplasmy

**Figure 10:**
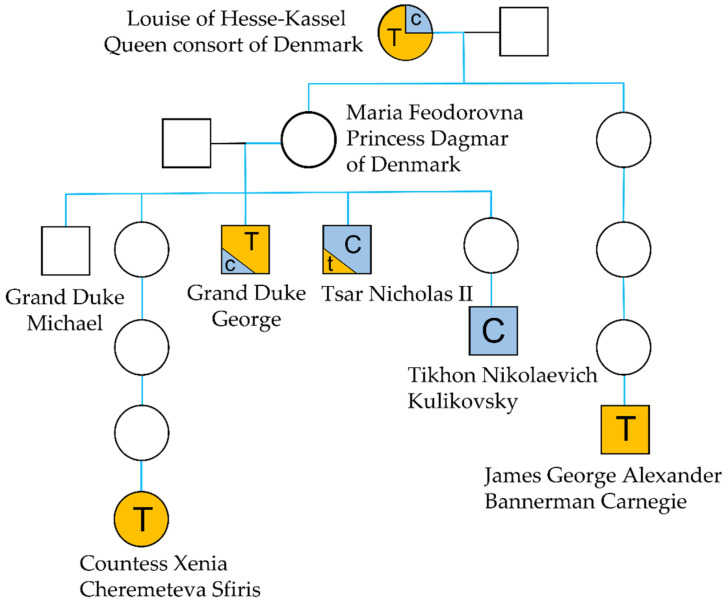
Family tree of the Romanov family showing the major allele (T or C) at heteroplasmic position 16169.

Mitochondrial DNA sequencing was performed to test the hypothesis that the frame hairs are from Tsar Nicholas’ maternal grandmother, Queen Louise of Hesse-Kassel. Fortunately, the mtDNA sequence of the Tsar’s maternal lineage is well documented. His control region (CR) haplotype was first reported in a study on the DNA analysis of skeletal remains that were discovered in Russia in 1991 and attributed to Nicholas II. The profile was compared to the CR of two living relatives who served as maternal references: James George Alexander Bannerman Carnegie, 3rd Duke of Fife (1929–2015), the Tsar’s first cousin twice removed and Countess Xenia Cheremeteva Sfiris (b. 1942), his great grandniece [3] (Figure 10). The profile of Tsar Nicholas revealed a C/T point heteroplasmy at position 16169 that was not present in the two references CR profiles that only had the 16169T variant. Since little was known about the occurrence of mitochondrial DNA heteroplasmy variants in the early 1990s, the mixed C/T position was originally viewed by skeptics as a “mismatch” between the tsar’s remains and his living relatives. However, two studies, both published in 1996, settled the debate. Ivanov et al. [4] analyzed the skeletal remains of the Tsar’s brother, Grand Duke George (1871–1899) and the study revealed once more, the presence of a point heteroplasmy at position 16169, albeit with opposite ratios. While C was the predominant base in Nicholas’ DNA, T was the dominant allele that was observed in his brother’s profile. Concurrently, the CR from Tikhon Nikolaevich Kulikovsky-Romanov (1917–1993), one of Tsar Nicholas nephews, was also Sanger sequenced and, in this case, only cytosines were observed at position 16169, adding credence to the presence of an heteroplasmic variant in the Tsar’s DNA [41].

The point heteroplasmy in the Tsar’s lineage was again confirmed in 2009, following the discovery of skeletal remains that were believed to be the two missing Romanov children. Additional genetic analyses of all the skeletal remains that were found in 1991 not only confirmed that the two sets of newly discovered skeletal remains were the two missing Romanov children [5], but also re-established the presence of point heteroplasmy in the Tsar’s remains [6].

In this study, the 16169 point heteroplasmy was not observed in skeletal remains, but in hair shafts. The occurrence of mtDNA heteroplasmy in human hairs has been extensively characterized [42,43]. Studies show that different levels of heteroplasmy can be observed in hairs from a single individual, as was seen in [44,45,46,47] and even along the length of a single hair shaft [48]. Our study followed the transmission of heteroplasmy through six generations. Interestingly, while it endured between the two generations that separate the Tsar from his grandmother, it seems to have rapidly evolved towards homoplasmy in the three different branches of the family that were studied. The perfect match between the sequence of the Tsar’s mtGenome and the sequence from the frame hairs, as well as the presence of a point heteroplasmy at position 16169 are consistent with the hairs coming from Louise of Hesse-Kassel, Queen of Denmark or another individual that is maternally related to Tsar Nicholas II.

## 6. Conclusions

We have previously shown that nuclear DNA can be recovered from single rootless hair shafts [12]. Here, we extend that work by demonstrating that both mitochondrial and nuclear DNA can be recovered from single rootless hairs of substantial age when an extraction protocol that is designed for highly fragmented DNA is employed. Further, by optimizing the extraction protocol to eliminate/reduce low level contaminating DNA from the reagents and supplies, we improved both DNA yields and downstream sequencing results.

Given that traditional nuclear and mitochondrial DNA typing assays generally target DNA fragments larger than 100–150 bp, extraction protocols optimized for the retention of smaller fragments may often be unnecessary. For the most compromised specimens, however, this type of specialized extraction coupled with shotgun sequencing, may offer the best and perhaps only chance of recovering probative DNA data. With an average mtDNA and nuclear DNA fragment sizes of approximately 40–85 bp and 33 bp, respectively, the hair samples tested here would have failed to produce DNA results with even the smallest mtDNA amplicons routinely used in operational casework. In addition, standard extraction protocols would likely have discarded much or most of the endogenous target DNA. By combining an optimized extraction protocol and next generation shotgun sequencing, it was possible to recover complete mtGenomes from hair samples that are most likely older than any evidence or reference hair sample that is generally submitted to a crime laboratory. These very old samples not only produced 14-times more mtDNA sequencing data than the control region would, they also produced enough nuclear DNA to assess the biological sex and, in one case, relatedness among samples.

Our results further demonstrate that probative nuclear DNA can likely be recovered from the types of single rootless hair shafts encountered in casework and generally tested for mtDNA only. By extension, the results suggest that aged hair from keepsake lockets, hairbrushes, and other aged sources may serve as alternative direct or familial reference material for missing persons cases that are requiring nuclear DNA and for which hair samples are the only available material. Finally, it is likely that this or similar extraction protocol(s) can be useful on any type of highly degraded sample. Indeed, we have also used this protocol for samples that were not hairs and can confirm that it can be extremely successful with any sample type that contains highly degraded DNA molecules.

## Figures and Tables

**Table 1:** Description of the hair shaft samples that were used in this study and purification methods that were used during DNA extraction.

Associated Item	Hair Shafts Tested	Experiment	Purification Protocol
	Single hair of ~5 cm (light brown). **Lo1a**	Microscopic examination	NA
	Single hair of ~2.5 cm (light brown). **Lo1b**	Microscopic examination	
	Single hair of ~3.8 cm (light brown). **Lo1c**	Microscopic examination	
**Fabergé Locket**	5 hairs for a total of ~16 cm. **Lo5h**	DNA	MinElute column
	Single hair of 4 cm (light brown). **Lo2**	DNA	Magnetic silica beads
	Single hair of 3.2 cm (light brown). **Lo3**	DNA	Magnetic silica beads
	Single hair of 6 cm (white). **Q3**	Microscopic examination	NA
**Frame**	6 hairs for a total of ~27 cm. **Q6h**	DNA	MinElute column
	Single hair of 5.5 cm (white). **Q1**	DNA	Magnetic silica beads
	Single hair of 7 cm (brown). **Q2**	DNA	Magnetic silica beads

**Table 2:** Sequencing statistics from the Fabergé locket hairs.

**Shotgun Libraries**	**Platforms Used**	**Number of Mapped Reads**	**Percentage of Mapped Reads**	**Number of Unique Reads**
Lo5h-sh (5 hairs)	MiSeq FGx^TM^	6,229,985	48%	5,500,712
Lo2-sh (1 hair)	NextSeq 500	199,948,284	66.54%	38,036,020
Lo3-sh (1 hair)	NextSeq 500	151,630,120	78.91%	33,388,550
**mtDNA-Enriched** **Library**	**Platforms Used**	**Number of Mapped Reads**	**Percentage of Mapped Reads**	**Number of Unique mtDNA Reads**
Lo5h-mt (5 hairs)	NextSeq 500	21,109,044	97.50%	35,188

**Table 3:** Average length of the mtDNA molecules in the locket libraries when mapped to hg19 (left) or to rCRS (right).

Library	Mapped mtDNA Reads Average Length (bp)
Lo2-sh	67.82/67.89
Lo3-sh	84.99/85.17
Lo5h-sh	66.04/69.16
Lo5h-mt	93.46

**Table 4:** Average length of the nuclear DNA molecules in the locket libraries.

Library	Mapped Nuclear DNA Reads Average Length (bp)
Lo2-sh	33.05
Lo3-sh	33.13
Lo5h-sh	31.96

**Table 5:** Determination of the biological sex of the donor of the hairs that were found in the Fabergé locket using Rx as described in [18]. SD: standard deviation. CI: Confidence interval. The values indicating a biological sex of female are in bold.

Library	Rx	SD	CI	CI Low	CI Sup
Lo2-sh	0.965	0.231	0.097	**0.869**	1.062
Lo3-sh	0.935	0.229	0.097	**0.838**	1.031

**Table 6:** Sequencing data from libraries made with DNA extracted from hair shafts found in the picture frame.

**Shotgun Libraries**	**Platforms Used**	**Number of Mapped Raw Reads**	**Percentage of Raw Mapped Reads**	**Number of Unique Human Reads**
Q6h-sh (6 hairs)	MiSeq FGx	2,072,278	21.17%	1,817,968
Q1-sh (1 hair)	MiSeq FGx	267,392	70.12%	239,692
Q2-sh (1 hair)	NextSeq 500	278,531,240	89%	56,390,706
**mtDNA-Enriched Libraries**	**Platforms Used**	**Number of Mapped Raw Reads**	**Percentage of Raw Mapped mtDNA Reads**	**Number of Unique mtDNA Reads**
Q6h-mt (6 hairs)	MiSeq FGx	7,059,775	90.68%	26,038
Q1-mt (1 hair)	MiSeq FGx	3,586,989	96.69%	14,514
Q2-mt (1 hair)	MiSeq FGx	8,104,414	88%	24,636

**Table 7:** Number of unique mitochondrial DNA reads and mean mtDNA lengths in the shotgun and capture libraries.

Sequencing Platform	Library	Number of Unique mtDNA Reads	Average mtDNA Size (bp). hg19/rCRS
MiSeq FGx^TM^	Q6h-sh	11,052	45.78/45.56
MiSeq FGx^TM^	Q6h-mt	26,038	47.26
MiSeq FGx^TM^	Q1-sh	1281	NA
MiSeq FGx^TM^	Q1-mt	14,514	38.80
NextSeq 500	Q2-sh	15,267	42.32/43.9
MiSeq FGx^TM^	Q2-mt	24,636	45.33

**Table 8:** Determination of the biological sex of the donor of two hairs that were found in the frame using Rx as described in [18]. SD: standard deviation. CI: Confidence interval. The values indicating a biological sex of female are in bold.

Library	Rx	SD	CI	CI Low	CI Sup
Q1-sh	0.903	0.201	0.084	**0.819**	0.987
Q2-sh	0.964	0.228	0.095	**0.868**	1.059

## Data Availability

The mtDNA data can be found in GenBank under project number PRJNA782999. For GenBank Biosample numbers, see Tables S2 and S3.

## Supplementary Materials

The following supporting information can be downloaded at: https://www.mdpi.com/article/10.3390/genes13020202/s1. Supplementary Material #1: Description of the optimized DNA extraction protocol for hair shafts; Supplementary Material #2: Description of the forced haploid and simulation method; Table S1: Sequencing statistics for all the hairs that were analyzed in this study. Table S2: Variant calls for the hairs found in the Fabergé locket. Table S3: Variant calls for the hairs found in the picture frame. Table S4: Data from reagent blanks. Table S5: Nuclear and mitochondrial DNA length observed in all the libraries. Figure S1: Pictures of the Fabergé locket. A. Locket and items that were recovered inside the right side of the jewel. B. Open locket showing the engraved number 15,650. C. Back of the locket showing the Tsarina’s initials and the imperial crown of Russia. D. Jump ring from the locket showing the stamped initials of Karl Fabergé. E. Lock of hairs that were found in the locket; Figure S2: Picture frame A. before and B. after restoration by Mr. Bachmakov; Figure S3: RAST results for Lo5h-sh. Figure S4: A. Cabinet card showing a photograph of Tsarina Alexandra made by Alexander Pasetti. B. Close up view of the Tsarina’s face. C. Picture that was found in the locket; Figure S5: Picture of Queen Louise of Hesse-Kassel taken in 1863 (source: Wikipedia). B. Back of the cabinet card found in the picture frame partially showing the name of Danish photographer Mary Steen. C. Front of the picture found in the picture frame.
