# Supplementary material for: Improved DNA Extraction and Illumina Sequencing of DNA Recovered from Aged Rootless Hair Shafts Found in Relics Associated with the Romanov Family
Source: Genes (Basel). 2022 Jan 23;13(2):202. doi: 10.3390/genes13020202 (PMC8872530; doi:10.3390/genes13020202)
Supplement: Supplementary file 1 [file genes-13-00202-s001.zip › Supplementary Figure S3.pdf]

Supplementary Figure S3

RAST Results for Lo5h-sh non-human reads

PHYLUM

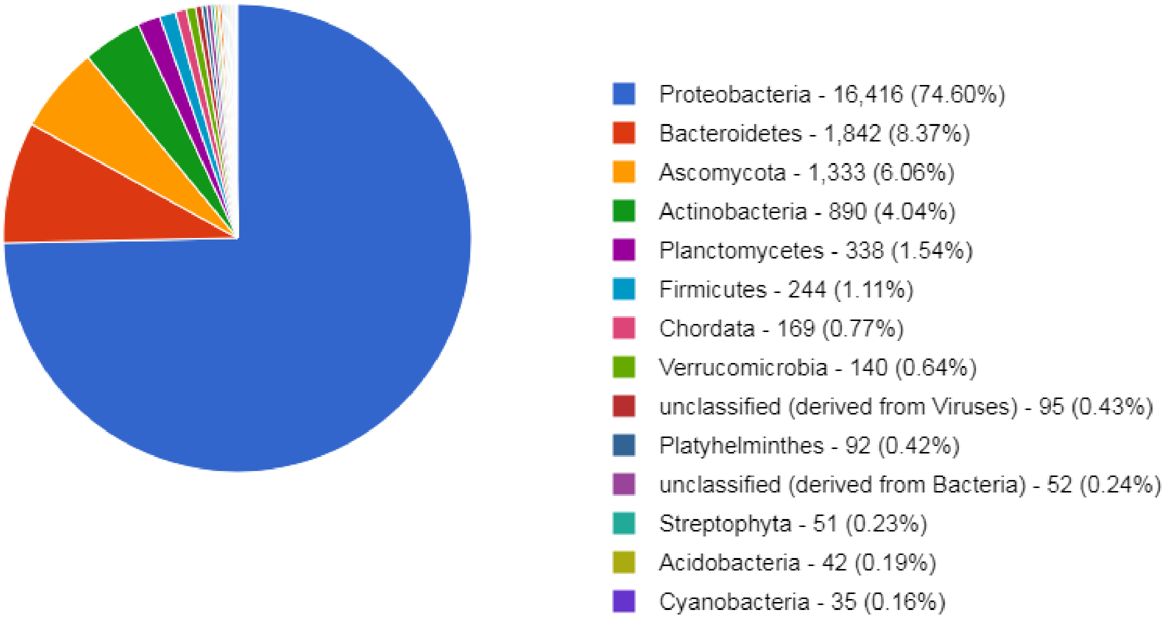

CLASS

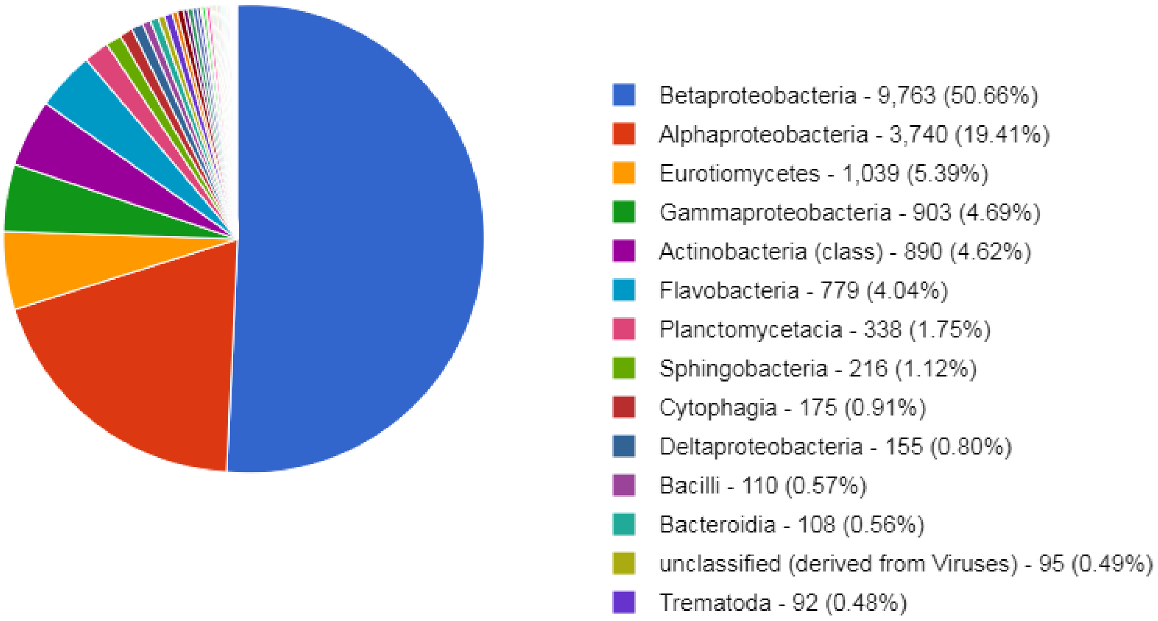

ORDER

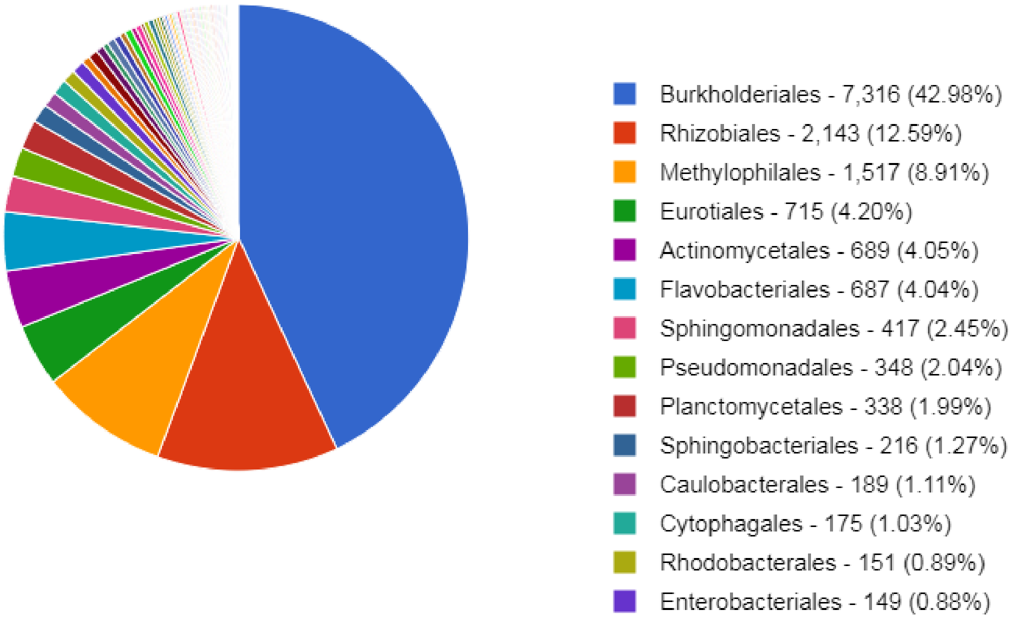

Burkholderiales - 7,316 (42.98%)
Rhizobiales - 2,143 (12.59%)
Methylophilales - 1,517 (8.91%)
Eurotiales - 715 (4.20%)
Actinomycetales - 689 (4.05%)
Flavobacteriales - 687 (4.04%)
Sphingomonadales - 417 (2.45%)
Pseudomonadales - 348 (2.04%)
Planctomycetales - 338 (1.99%)
Sphingobacteriales - 216 (1.27%)
Caulobacterales - 189 (1.11%)
Cytophagales - 175 (1.03%)
Rhodobacterales - 151 (0.89%)
Enterobacteriales - 149 (0.88%)
