# Supplementary material for: Improved DNA Extraction and Illumina Sequencing of DNA Recovered from Aged Rootless Hair Shafts Found in Relics Associated with the Romanov Family
Source: Genes (Basel). 2022 Jan 23;13(2):202. doi: 10.3390/genes13020202 (PMC8872530; doi:10.3390/genes13020202)
Supplement: Supplementary file 1 [file genes-13-00202-s001.zip › Supplementary Material #1 11042021.pdf]

Supplementary Material

New DNA Extraction Protocol for Aged Hair Shafts

by Loreille O. et al.

This protocol should be carried out in a laboratory dedicated to low quantity and low quality DNA samples with limited personnel access. Recommended personal protective equipment used to minimize the risk of contamination must include Tyvek® IsoClean® single-use garments (frocks, sleeves, masks), goggles and double gloving. Reagents should be aliquoted upon arrival at the laboratory and all plastic consumables must be irradiated in a cross-linker for 30 minutes prior to use.

1. Decontamination of the hairs

- Place each hair sample in a 1.5 ml tube and submerge it with a 5% Terg-a-zyme solution (Alconox, New-York, NY).
- Sonicate for 20 minutes.
- Transfer the hair to a tube containing 1 ml of ethanol and mix to rinse.
- Transfer the hair to a tube containing 1 ml of DNA-free water and mix to rinse.

Note 1: sample is generally transferred from the last cleaning tube to a new tube containing the digestion buffer; however, the sample may remain in the same tube with the removal of the water rinse prior to the addition of the digestion buffer.

2. Lysis/Digestion for four samples and one reagent blank

- Prepare the digestion buffer by adding 26.4 µL of DTT (5M) to 2 ml of ATL Buffer (Qiagen, Germantown, MD). Vortex and irradiate in a cross-linker for 30 minutes.
- For each sample and RB, transfer the hair to a tube containing 300 µl of the digestion buffer and add 20 µL of proteinase K (20mg/ml) in each tube.
- Vortex and ensure that all samples are submerged.

Note 2: samples may be cut into pieces to ensure a full immersion.

- Incubate all the tubes in a thermomixer set at 56°C and 900 rpm for a minimum of 30 minutes, or until the hair is fully digested. Alternatively, samples can be incubated overnight in a thermomixer set at 37°C and 900 rpm.

Note 3: if full digestion does not occur, a decontaminated glass pestle can be used to break down the hair. Alternatively, extra proteinase K can be added, and the sample incubated again.

3. DNA purification for four samples and one reagent blank

- Binding buffer recipe for 5 samples/RB: 16 ml PB Buffer (Qiagen), 480 µl Sodium Acetate (3M) and 40 µl of Sodium Chloride (5M).

Note 4: the volume of binding buffer should always be 10 to 13 times the volume of the lysate.

- Add 3.5 ml of binding buffer into five irradiated 5 ml or 15 ml tubes.
- Once the hairs are fully digested, transfer the lysate to the tubes containing the binding buffer.
- Tighten the caps, wrap the top of each tube with parafilm. Vortex.
- Place the tubes flat on a nutator and turn the nutator on, for **an end-over-end gentle agitation**.
- Incubate/mix all five tubes for at least 1 hour at room temperature.
- During incubation, wash the magnetic beads (#786-915, G-Biosciences, Maryland Heights, MO).
  - Vortex/shake the bottle to resuspend the beads in the solution.
  - Using a low retention tip, pipette 75 µl of beads and transfer into a 1.5 ml low binding tube.
  - Spin very briefly and place the tube on a magnet such as a DynaMag™- 2 (Thermo Fisher Scientific, Waltham, MA) for 30-60 seconds.
  - Wait until the beads are immobilized on the tube wall.
  - Discard the supernatant by aspiration with a pipette.
  - Add 500 µl of EB Buffer (Qiagen).
  - Vortex the tube for 5-10 seconds, spin briefly and place the tube back on the magnet.
  - Discard the EB Buffer by aspiration with a pipette.
  - Repeat the last three steps twice for a total of 3 washes.
  - Discard all remaining EB Buffer.
  - Resuspend beads in 75 µl of EB Buffer.
  - Optional: using a low retention tip, transfer 15 µl of beads into five 1.5 ml low binding tubes. Make sure all the tubes contain the same volume of beads. Do not use expired beads.

- Once the incubation of the samples and RB is over, remove the parafilm and spin the tubes briefly to remove any solution in caps.
- Use low retention tips and mix the beads by pipetting up and down to homogenize.
- Transfer 15 μl of washed beads into each of the five tubes containing samples or RB.
- Tighten the caps and wrap the top of each tube with parafilm.
- Vortex for 5 seconds to homogenize the solution.
- Place the tubes flat on a nutator and turn the nutator on, for an **end-over-end gentle agitation**.
- Incubate/mix the tubes at room temperature for at least 30 minutes.
- Once the second incubation is over, remove the parafilm and centrifuge all the tubes to remove any solution in caps.
- Take the first tube, loosen the cap and place it on a DynaMag^{TM}-15 Magnet (Thermo Fisher Scientific).
- Wait until the beads are immobilized on the tube wall.
- Using a 1000 μl pipette, transfer 3 ml of solution to a newly labeled tube (henceforward referred to as: ** tubes).
- Remove the tube containing the beads and ~ 520 μl of solution from the magnet.
- Vortex until the beads have detached from the tube wall and are fully resuspended in the solution.

<u>Note 5</u>*: do not centrifuge the tubes but tap them gently to get all the beads and liquid down*.

- Transfer the beads in solution to a new labeled 1.5 ml DNA-free, low binding tube.
- Wipe the 1000 μl pipette thoroughly with an isopropanol wipe.
- Repeat process for the other samples and RB.
- Place all 1.5 ml tubes on a DynaMag^{TM}- 2 magnet and wait until all the beads are immobilized on the tube walls.
- Transfer the remaining solution to the **tubes and store these at +4°C or -20°C.
- Dispense 500 μl of Buffer PE (Qiagen) to each tube.
- Vortex all the tubes for 5-10 seconds, spin briefly and place the tubes back on the magnet.
- Wait until the beads are immobilized and discard the PE.
- Repeat the last three steps twice for a total of 3 washes.
- After the 3rd wash, remove as much PE Buffer as possible without disturbing the beads.
- Air dry the beads in the hood for ~10 minutes. Do not over dry them.
- For each sample and RB: take the tube off the magnet and add 50 to 100 μl EB Buffer (or TLE) to the beads pellets; pipette up and down to resuspend the beads in the solution.

- Close the lid and incubate at room temperature for 5 minutes.
- Vortex briefly, spin and place the tubes back on the magnet; wait for all the beads to be immobilized on the tube wall.
- Collect the extracts and transfer to new labeled DNA-free 1.5 low binding tubes.
- Place all the extracts back on the magnet and wait for at least 3 minutes.
- If some beads are still visible, collect the extracts a second time.
- Freeze the extracts and RB at -20°C or proceed to the next step.

Note 6: If the DynaMag^{TM}-15 is not available, it is possible to use a DynaMag^{TM}-2 and tape the tubes against the magnet.

Note 7: if the samples are small and/or the DynaMag^{TM}-15 is not available, use a 2 ml DNA-free tube and lyse the hair in 100 μl of digestion buffer + 10 or 20 μl of proteinase K. When the hair digestion is over, add 1.5 ml of binding buffer to the tube with the lysate, vortex and incubate at room temperature. Then follow the protocol described above.
