# Supplementary material for: Improved DNA Extraction and Illumina Sequencing of DNA Recovered from Aged Rootless Hair Shafts Found in Relics Associated with the Romanov Family
Source: Genes (Basel). 2022 Jan 23;13(2):202. doi: 10.3390/genes13020202 (PMC8872530; doi:10.3390/genes13020202)
Supplement: Supplementary file 1 [file genes-13-00202-s001.zip › Supplementary Material #2.pdf]

Supplementary Material #2

Description of the forced haploid and simulation method

Introduction

In this study, one task was to determine whether or not different hair samples originate from the same individual, from close relatives or from unrelated individuals. Since only very low coverage autosomal DNA data sets were available, heterozygous genotypes could not be called accurately. Such low coverage data does, however, still contain information about the degree of relationship, based on allele sharing proportions. Different approaches to estimate the degree of relationship from low coverage DNA data sets have previously been developed [e.g., Fernandes et al., 2017; Martin et al., 2017, Monroy et al., 2018,]. While our approach has several similarities to these, there are also differences. Instead of estimating a kinship coefficient parameter as a metric to infer the degree of relatedness, we measured the allele mismatch proportion between pseudo-haploid genomes of the tested samples and then combined this with a simulation approach to obtain expected mismatch distributions for various degrees of relatedness (see Table S6, S7 and Figure S7 for an illustration). Such mismatch proportions depend not only on the degree of relatedness, but also on the heterozygosity of the chosen SNPs (i.e., allele frequency distributions). By using SNP specific population allele frequencies, specified pedigrees, and an error model to account for possible genotype errors in the datasets, our simulation model produced estimates from which the degree of relatedness could be inferred.

SNP (alleles in the population)	Observation (coverage)	Possible true genotype given the observation and population data	Comment
SNP 1 (A/G)	A (coverage = 1)	A/A or A/G	Allele drop-out?
SNP 2 (C/T)	C (coverage = 2)	C/C or CT	Allele drop-out?

Table S6. Illustrative example of the problem

SNP (alleles in the population)	True genotype ("in vivo")		Observation (coverage)		Forced pseudo-haploid genome data set	
	Sample 1	*Sample 2*	*Sample 1*	*Sample 2*	*Sample 1*	*Sample 2*
SNP 1 (A/G)	A/G	A/G	A (coverage = 1)	G (coverage = 1)	A	G
SNP 2 (C/T)	C/C	C/C	C (coverage = 2)	C (coverage = 2)	C	C
					Mismatch proportion = 1/2	

Table S7. Illustrative example of our approach

Material & Methods

Our approach to infer the degree of relatedness between the tested hair samples, Lo2 and Lo3, is built on the estimation of allele mismatch proportions. For each of the samples, we first targeted approximately 1.3 million autosomal SNPs [Tillmar et al, 2020, Tillmar et al., 2021] from the shotgun data. We then excluded SNPs that did not have any calls in either of the samples or in only one of the samples. The remaining SNP calls (i.e., SNPs with allele calls in both samples) were used as the input in our forced haploid and simulation method. In this method, the genotype data was initially forced into pseudo-haploid genome data sets, from which the proportion of mismatches was calculated. To obtain expected mismatch proportion distributions, we simulated pseudo-haploid datasets for various degrees of relatedness (i.e., same individual, parent/child, full siblings, and unrelated individuals) based on European allele frequencies [Genomes Project Consortium et al., 2015, via SNP-nexus (www.snp-nexus.org), Oscanoa et al., 2020] for the specific set of SNPs included in the sample comparisons. In addition, we included a simplistic error model to account for the possibility of different genotype errors (e.g., deamination, depurination, PCR error, sequencing error and mapping error) that may occur for low quality and low quantity DNA samples. We let the error rate vary between 0 and 2%. See Figure S7 for more information about the simulation method.

As controls, we analyzed and compared two hair samples, 189A and 189B, which were known to originate from the same individual. We also compared shotgun data from 189A with data from Lo2 which were known to originate from two unrelated individuals.

Simulation method
(exemplified for siblings and 2 SNPs)

Population data (allele frequencies)

	A	C	G	T
SNP 1	0.3	0	0.7	0
SNP 2	0	0.1	0	0.9

1. Draw alleles for founders
   (incl. chromosomal phasing)

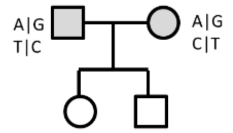

2. Allele dropping
   (incl. crossing-over events (recombination))

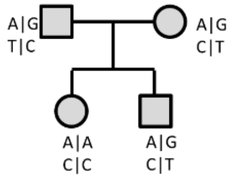

3. Remove alleles for untyped individuals, and remove the phase

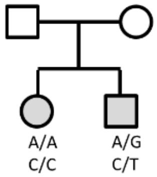

4. Forced the genotype data into pseudo-haploid genomes

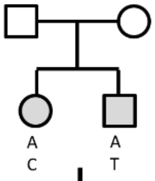

5. Introduce genotype errors
   (no errors in this example)

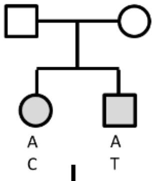

6. Estimate the proportion of mismatches between the different individuals/samples

$$\text{Mismatch proportion} = \frac{1}{2} = 0.5$$

7. Repeat steps 1-6, say 1000 times, and plot the distribution of the «mismatch proportion»

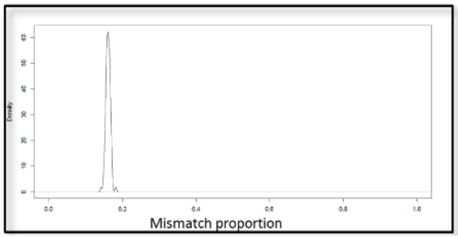

The result is the distribution of the expected mismatch proportion for the chosen relationship, set of SNPs, allele frequencies and error rate.

Figure S7. Our forced haploid and simulation approach. The simulation method was used to obtain expected mismatch proportions for various degrees of relatedness based on included SNP markers, their allele frequencies and genotype error rate.

Results

The result from the comparison between Lo2 and Lo3 is described in the main article. The results from the control experiments are shown in Figure S8. The comparison between 189A and 189B resulted in 55,915 SNPs which gave allele calls for both samples. After forcing these datasets into pseudo-haploid genomes, the mismatch proportion was calculated to be 0.13. Such a proportion was consistent with the expected mismatch distribution for same individuals and fell outside the expected proportions for parent/child, full siblings, and unrelated individuals when the error rate was set to 0. This result matches the expectation since 189A and 189B were known to originate from the same individual. For larger error rates, the observed mismatch proportion was smaller than the expected mismatch distributions, even for the same individuals. Samples 189A and 189B were however both of better quality than the Lo2 and Lo3 samples, and the genotype error rate is expected to be lower for 189A and 189B.

The comparison between 189A and Lo2 resulted in 4,515 SNPs which gave allele calls for both samples. After forcing these datasets into pseudo-haploid genomes, the mismatch proportion was calculated to be 0.20. Such a proportion was consistent with the expected mismatch proportion for unrelated individuals (for the error rates equal to, or less, than 1 %) (Figure S8). For the simulations performed with an error rate of 2 %, the observed proportion fell outside the expected mismatch distribution for all relationships included in this study. Sample 189A was of relatively good quality, however, and thus the genotype error rate in this comparison was expected to be close to the 1% or less.

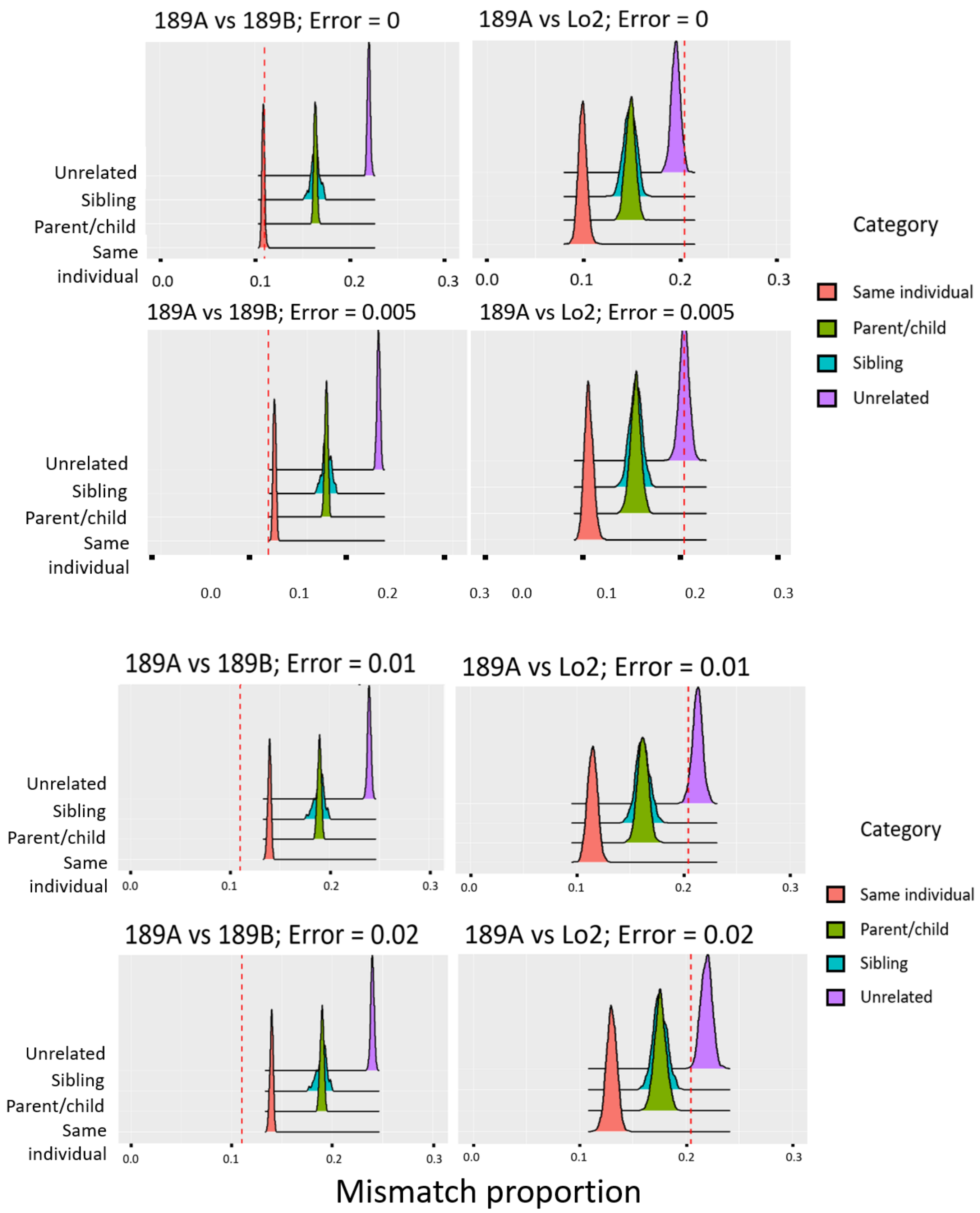

189A vs 189B; Error = 0
189A vs Lo2; Error = 0
189A vs 189B; Error = 0.005
189A vs Lo2; Error = 0.005
189A vs 189B; Error = 0.01
189A vs Lo2; Error = 0.01
189A vs 189B; Error = 0.02
189A vs Lo2; Error = 0.02
Unrelated
Sibling
Parent/child
Same individual
0.0
0.1
0.2
0.3
Category
Same individual
Parent/child
Sibling
Unrelated
Mismatch proportion

Figure S8. Observed and expected mismatch proportions for the 189A vs 189B comparison (left), and for the 189A and Lo2 comparison (right).

Genotype error rate estimation

In order to estimate the error rate for Lo2 and Lo3 we specifically checked SNPs assumed to be fixed in the population (i.e., SNPs with a minor allele frequency < 0.1%, based on the 1000 Genomes data). Under the assumption that a variant call at these SNPs represents a genotype error, the rate was estimated at approximately 1 % for Lo2, and 1.8 % for Lo3. For the control samples 189A and 189B, the genotype error rates were estimated to be around 0.5%. Though these error rate estimates were based on relatively few observations, and should thus be interpreted with care, they nevertheless provide aa gauge of the magnitude of error for the different samples.

References

Fernandes, D.; Sirak, K.; Novak, M.; Finarelli, J.A.; Byrne, J.; Connolly, E.; Carlsson, J.E.; Ferretti, E.; Pinhasi, R.; Carlsson,J. The identification of a 1916 Irish rebel: new approach for estimating relatedness from low coverage homozygous Genomes. *Sci Rep*. **2017**,*7*,41529.

Martin, M.D.; Jay, F.; Castellano, S.; Slatkin, M. Determination of genetic relatedness from low-coverage human genome sequences using pedigree simulations. *Mol. Ecol*. **2017**, *26*, 4145-4157.

Monroy Kuhn, J.M.; Jakobsson, M.; Gunther, T. Estimating genetic kin relationships in prehistoric populations. *PLoS One* **2018**,*13*, e0195491.

Tillmar, A.; Fagerholm, S.A.; Staaf, J.; Sjölund, P.; Ansell, R. Getting the conclusive lead with investigative genetic genealogy - A successful case study of a 16-year-old double murder in Sweden. *Forensic Sci Int Genet.* **2021**, *53*,10252.

Tillmar, A.; Sjölund, P.; Lundqvist, B.; Klippmark, T.; Älgenäs, C.; Green, H. Whole-genome sequencing of human remains to enable genealogy DNA database searches - A case report. *Forensic Sci Int Genet*. **2020**, 46, 102233.

1000 Genomes Project Consortium; Auton, A.; Brooks, L.D.; Durbin, R.M.; Garrison, E.P.; Kang, H.M.; Korbel, J.O.; Marchini, J.L.; McCarthy, S.; McVean, G.A.; Abecasis, G.R. A global reference for human genetic variation. *Nature* **2015**, *526*, 68-74.

Oscanoa, J.; Sivapalan, L.; Gadaleta, E.; Dayem Ullah, A.Z.; Lemoine, N.R.; Chelala, C. SNPnexus: a web server for functional annotation of human genome sequence variation (2020 update). *Nucleic Acids Res*. **2020**, *48*, W185-W192.
