# Supplementary figures and images for: Improved DNA Extraction and Illumina Sequencing of DNA Recovered from Aged Rootless Hair Shafts Found in Relics Associated with the Romanov Family
Source: Genes (Basel). 2022 Jan 23;13(2):202. doi: 10.3390/genes13020202 (PMC8872530; doi:10.3390/genes13020202)

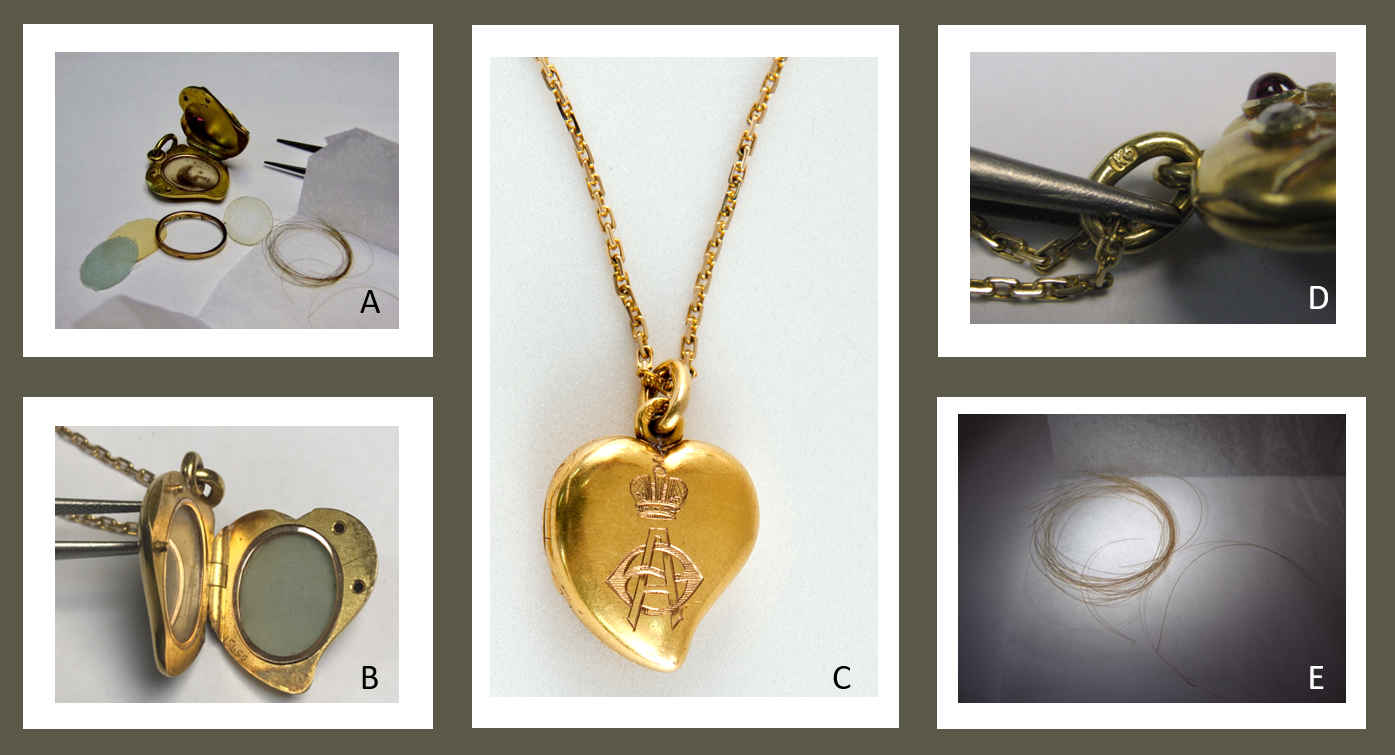

Supplement: Supplementary file 1 [file genes-13-00202-s001.zip › Supplementary Figure S1.PNG]

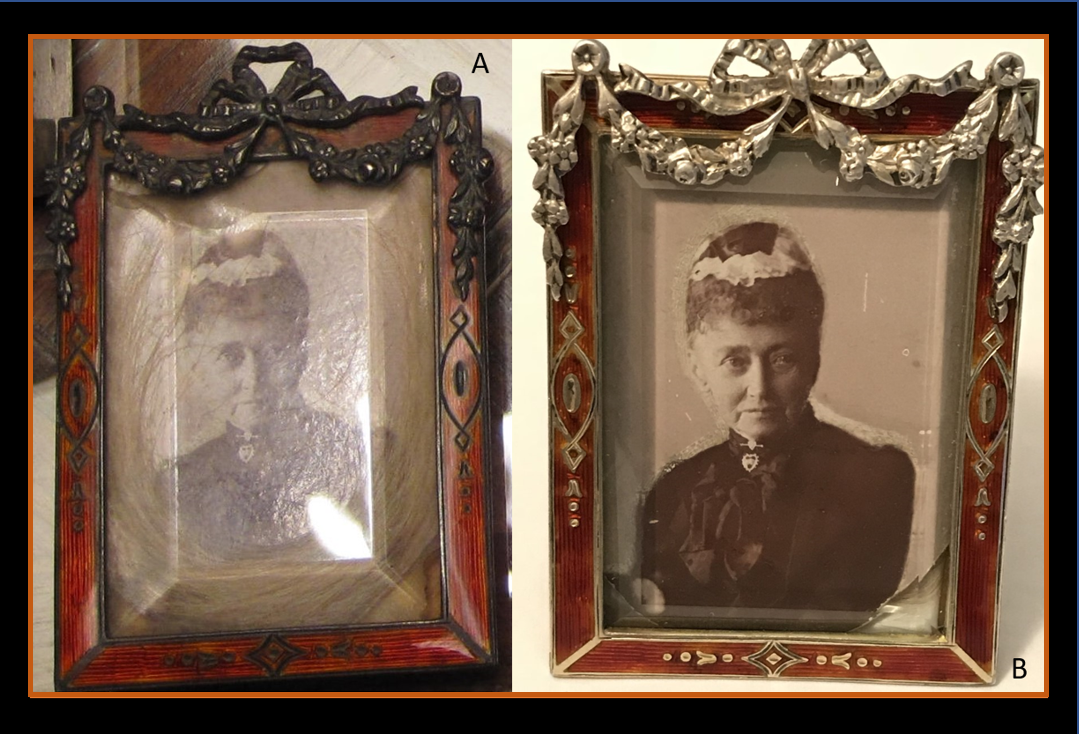

Supplement: Supplementary file 1 [file genes-13-00202-s001.zip › Supplementary Figure S2.PNG]

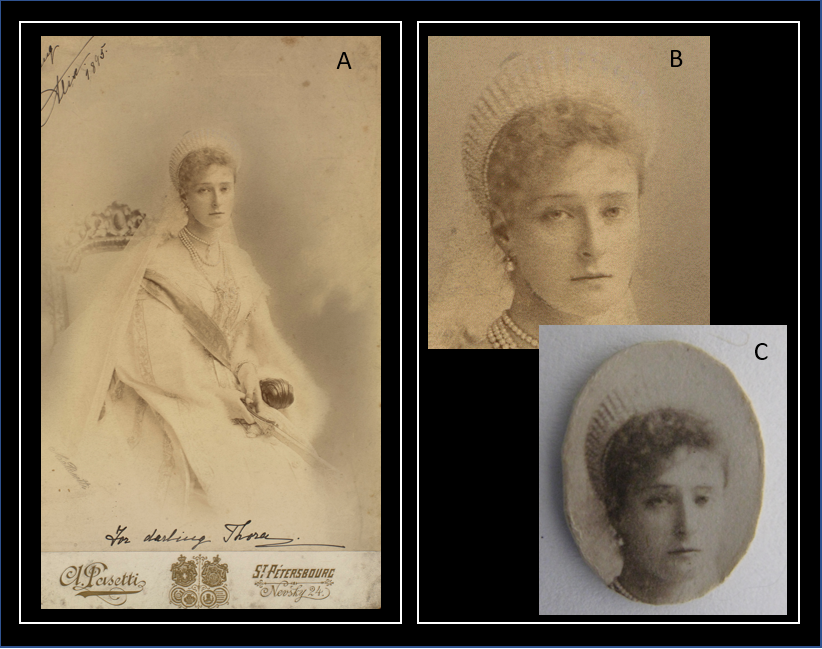

Supplement: Supplementary file 1 [file genes-13-00202-s001.zip › Supplementary Figure S4.PNG]

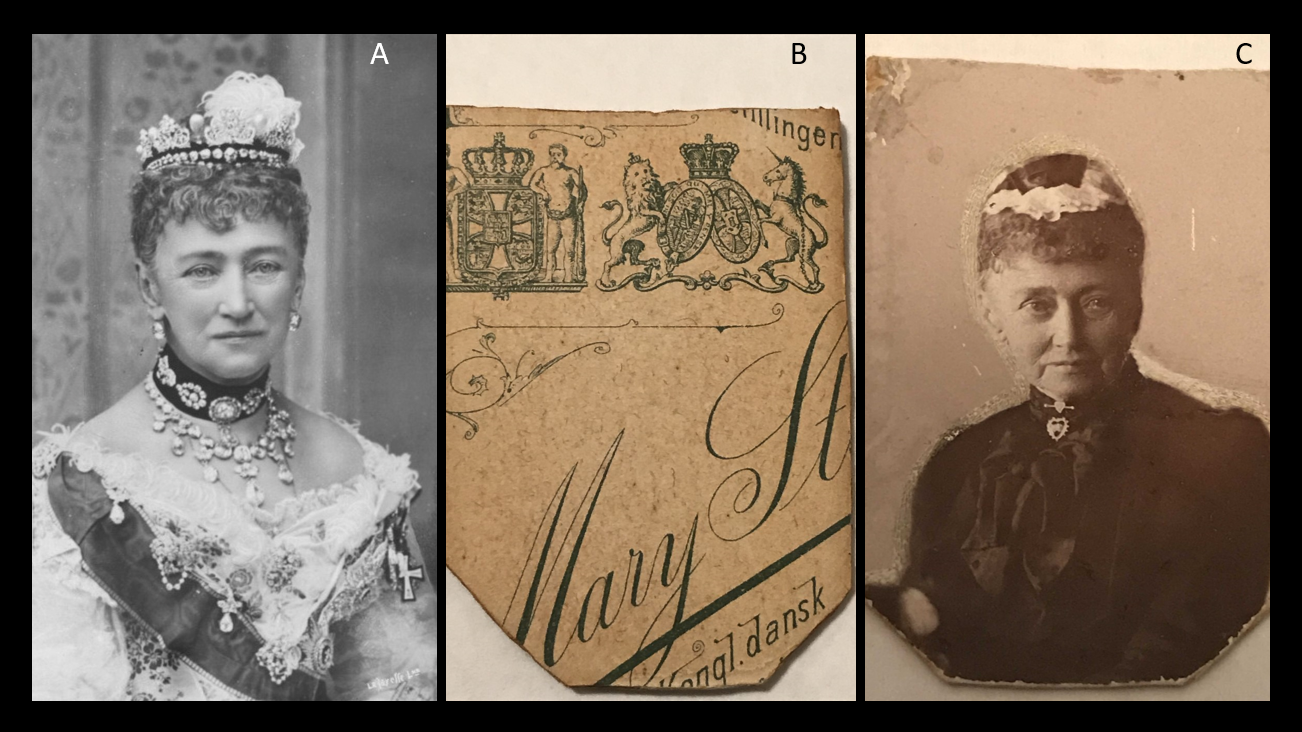

Supplement: Supplementary file 1 [file genes-13-00202-s001.zip › Supplementary Figure S5.PNG]
